# Translation and cross-cultural adaptation of the Brazilian version of the Down Syndrome Speech Intelligibility Survey

**DOI:** 10.1590/2317-1782/e20240079en

**Published:** 2025-03-31

**Authors:** Julyane Feitoza Coêlho, Gustavo Lopez Estivalet, Francisco Tiago Meireles da Silva, Isabelle Cahino Delgado, Leandro de Araújo Pernambuco, Giorvan Ânderson dos Santos Alves

**Affiliations:** 1 Programa de Pós-graduação em Linguística, Universidade Federal da Paraíba – UFPB - João Pessoa (PB), Brasil.; 2 Departamento de Letras Estrangeiras Modernas, Universidade Federal da Paraíba – UFPB - João Pessoa (PB), Brasil.; 3 Departamento de Fonoaudiologia, Universidade Federal da Paraíba – UFPB - João Pessoa (PB), Brasil.; 4 Departamento de Fonoaudiologia, Universidade Federal de Pernambuco – UFPE - Recife (PE), Brasil.

**Keywords:** Down Syndrome, Apraxias, Mass Screening, Diagnosis, Diagnosis, Differential

## Abstract

**Purpose:**

Translate and cross-culturally adapt the “Down Syndrome Speech Intelligibility Survey” questionnaire into Brazilian Portuguese.

**Methods:**

The following steps were taken for cross-cultural adaptation: translation of the instrument from the source language to the target language, synthesis of the translated versions, evaluation of the synthesis by expert judges, evaluation of the instrument by the target audience, back translation, and pilot study.

**Results:**

After the translation and synthesis of the translations, most expert judges analyzed almost all instrument items (n = 44, 97.77%) as very relevant, very feasible, and appropriate. In the analysis by the target population, parents suggested changes, improving the understanding of the instrument. The back translation revealed some inconsistencies in the translated and adapted version in relation to the original instrument’s content, and the appropriate adjustments were made. The pilot study identified the need to adapt some questions, exclude one item (which had similar content to another), and include prior instructions for completion, resulting in the final instrument version.

**Conclusion:**

The translation and cross-cultural adaptation verified the validity evidence based on the content of the Brazilian version of the Down Syndrome Speech Intelligibility Survey.

## INTRODUCTION

Down syndrome is a genetic condition characterized by a change in the distribution of chromosomes in cells, typically involving an extra chromosome in the 21st pair^(1)^. In this context, there is a current preference for using the term Trisomy 21 (T21).

T21 has several implications for global development, including linguistic and communicative impairments. These deficits are associated with cognitive, motor, auditory, and oral myofunctional system development. Regarding specific language delays and challenges, various impairments have been found over time in individuals with this condition, particularly in phonological processing and syntactic development. A review study indicated that individuals with T21 may face specific challenges with phonological processing due to pronounced deficits in short-term verbal memory^(2)^.

The speech of individuals with T21 may be altered due to the characteristics of their stomatognathic system, as well as difficulties in programming movements and sequences necessary for speech sound production. Speech apraxia is clinically characterized in these individuals when their ability to voluntarily program speech movements is impaired, associated with reduced speech intelligibility, inconsistent errors, and difficulties in sequencing sounds and oral movements^(3)^.

Childhood apraxia of speech (CAS) is a neurological speech sound disorder that occurs in childhood, where the precision and consistency of the movements underlying speech are impaired in the absence of neuromuscular deficits. CAS may occur because of known neurological impairments; in association with complex neurobehavioral disorders of known or unknown origin; or as an idiopathic neurogenic speech sound disorder. The central impairment in planning and/or programming the spatial-temporal parameters of movement sequences results in errors in speech sound production and prosody^(4)^.

This condition has been identified in individuals with T21 with a prevalence of 11.1%, based on the analysis of speech samples from 45 participants aged 10 to 20 years, using perceptual and acoustic methods and measures from the Speech Disorders Classification System (SDCS). It was also found that nearly the entire sample of participants with T21 had some type of speech sound disorder and motor speech disorder^(5)^.

One of the challenges in this context is the differential diagnosis of speech disorders. Hence, the speech-language-hearing assessment of language in T21 involves a multi-step investigation, addressing both expressive and receptive aspects. Language analysis covers various linguistic levels and may also involve, from a multimodal perspective, analysis of the effective use of gestures and facial expressions to aid communication to identify the greatest impairments and potentialities, guide intervention, and stimulate the development of linguistic and communicative skills.

Coexisting impairments, combined with the intellectual deficit characteristic of the syndrome, require a detailed investigation and complicate the differential diagnosis of speech disorders. Hence, the lack of instruments in Brazilian Portuguese validated for use with this population poses further challenges.

It is significantly relevant to screen for CAS in T21, considering the recommendation to subject the population to specific tests based on the epidemiological aspects of the condition^(6)^. Screening identifies the condition's characteristics, which is prevalent in this specific population^(5)^. Thus, it is crucial to use instruments to screen and identify individuals with signs suggestive of CAS and require targeted evaluation for the disorder.

Therefore, this study aimed to help investigate speech apraxia associated with this genetic condition in Brazilians with T21, by conducting a cross-cultural adaptation of the Down Syndrome Speech Intelligibility Survey^(7)^. This instrument was developed for application with parents of individuals with T21 and is considered a useful tool for screening this motor speech disorder^(8)^.

Cross-cultural adaptation creates equivalent versions of an instrument translated into a new language, facilitating cross-cultural studies and the use of previously developed and validated tools. It is carried out through a specific methodology with several stages, such as translating the instrument from the source language to the target language, synthesizing the translated versions, having expert judges evaluate the synthesis and the target population assess the instrument, back translating, and pilot testing^(9)^.

This instrument was chosen for cross-cultural adaptation because it is the only one available for investigating these characteristics specifically in this population. This questionnaire is highly relevant and was used for the first broad survey on the characteristics of speech apraxia in the T21 population. The instrument has already been translated and adapted into Turkish, with promising results, and has been identified as a screening tool for a possible diagnosis of speech apraxia associated with T21^(8)^.

Additionally, the need to adapt this instrument is justified by the absence of any questionnaire in Portuguese designed to analyze these aspects, specifically for use with parents of individuals with T21 – an important audience for identifying these speech characteristics.

Thus, its cross-cultural adaptation and subsequent validation may contribute to the clinical practice of Brazilian speech-language-hearing pathologists, serving as a screening tool to assist in identifying individuals who require specific evaluation for the differential diagnosis of speech disorders.

Thus, this study aimed to translate and cross-culturally adapt the Down Syndrome Speech Intelligibility Survey into Brazilian Portuguese.

## METHODS

The study considered all ethical aspects, in accordance with Brazilian Resolution no. 466/12^(10)^. The research project was previously submitted to the University’s Research Ethics Committee, where it was reviewed and approved through evaluation report number 5.127.264. During data collection, an informed consent form was presented to the participants, ensuring the confidentiality of all collected data. Additionally, prior authorization was obtained from the author of the original instrument for its cross-cultural adaptation.

### Instrument

The Down Syndrome Speech Intelligibility Survey^(7)^ was the instrument translated and adapted in this study. It is a questionnaire with five questions and 40 sentences, intended for use with parents of individuals with T21. In the items corresponding to the sentences, the informant must rate the frequency with which a particular characteristic is present in their child's speech (always, frequently, sometimes, or never). Thus, speech-language-hearing pathologists interpret the findings qualitatively.

### Data collection procedures

This research was conducted from September 2021 to August 2023. The cross-cultural adaptation process followed the methodology proposed by Borsa, Damasio, and Bandeira (2012), due to its feasibility and applicability. This methodological model includes six stages: (1) translation of the instrument from the source language into the target language, (2) synthesis of the translated versions, (3) evaluation of the synthesis by expert judges, (4) evaluation of the instrument by the target population, (5) back translation, and (6) pilot study.

Two translators fluent in both English and Portuguese initially translated the instrument. One of them is a speech-language-hearing pathologist and foreign language teacher, with knowledge of the subject matter, which helped in addressing the specific technical terms in the instrument's content. The other translator was a foreign language teacher, who focused on the linguistic characteristics and the more general aspects of the translation (e.g., the correspondence between the original and translated versions) and the instrument’s comprehension level.

After this stage, the research team (which included the two translators, the lead researcher, and the study supervisor) synthesized the two translated versions of the instrument, along with the original instrument. At this point, both translated versions were considered, and the team discussed which one best suited each item or made adjustments by combining both translations.

Next, the instrument was presented to a group of 17 speech-language-hearing pathologists with expertise in the field, invited to participate via email. Of these, 10 judges who volunteered to participate evaluated each item’s relevance, feasibility, and adequacy. The evaluation used a 4-level response scale, ranging from the absence of the characteristic to its highly intense presence (e.g., irrelevant, slightly relevant, relevant, and highly relevant). Nine women and one man participated in this stage, of whom six held a doctorate and four held a master's degree.

The following study stage applied the instrument to 14 parents of individuals with T21 – 13 mothers and one father, with diverse profiles. The goal was to test the translated instrument’s semantics and comprehension level. In this stage, the parents were asked to indicate whether they understood each item, its instructions, and the response scale. They could also, if necessary, suggest changes to the text to make it more suitable to their vocabulary.

To this end, the instrument was presented to the parents through an online questionnaire on Google Forms, sent via social media (messaging application), along with a video providing instructions on the analysis procedures. Nine parents participated in this stage, but only two of them were included as they properly completed the analysis in the online format, as instructed.

Data were also collected in person due to the parents’ difficulty in completing the analysis online. Hence, 12 parents were gathered in small groups and instructed on the procedures for analyzing the printed questionnaire. The sample was selected for convenience, inviting parents from an outreach program in the area, whose children were of the appropriate age for the instrument’s application (ranging from 1 to 21 years old).

Then, a native English speaker and Portuguese speaker, a professional translator, back translated the instrument from Portuguese into English without having access to the original instrument. Next, the translated and the back translated versions were compared with the original version, resulting in the adaptation of certain aspects of the protocol, and leading to the pre-final version of the translated and adapted instrument.

Finally, this last instrument version was presented to the original author, along with the back translated version, for approval. The research team performed a comparative analysis of the translated and adapted version with the back translated version.

After making the necessary adjustments, the version was applied to parents of individuals with T21 in a pilot study with participants from an outreach program and members of a nonprofit organization, both focused on individuals with T21. The aim was to assess whether the items were appropriate regarding their meaning, level of comprehension, and instructions for administration.

This final stage was conducted online, sharing the instrument via a messaging application, and sending it for completion through Google Forms. In this stage, 18 parents responded to the instrument, but two were excluded because their children were not in the established age range, and another two were excluded because they had already participated in the study during the target population analysis stage, serving as judges. Therefore, 14 parents were included, consisting of 13 mothers and one father.

These stages were primarily carried out online, with emails sent to the participating judges, forms filled out online, and videoconferences for consensus meetings among the researchers. Data were collected in person during the target population’s instrument analysis due to the sample’s difficulties in completing it online. This was held at the Speech-Language-Hearing Clinic of a University in João Pessoa, Paraíba, Brazil.

### Data analysis procedures

A qualitative and descriptive analysis of the entire translation and adaptation process was performed, encompassing all stages. A quantitative analysis was also performed in some study stages to consolidate the judgment results by the experts and parents and the pilot study results, grouping them into percentages. Additionally, an item content validity analysis was performed specifically during the expert committee’s judgment.

Content validity measures the degree of relevance and representativeness of the elements in a measurement instrument for a specific evaluative construct. Hence, judges analyze and assess the tool based on a developed instrument. The Content Validity Index (CVI) includes an overall analysis (Total CVI) and item-specific analysis (Item CVI – I-CVI) of the percentage/proportionality of the judges' opinions, with rates not lower than 0.80 or 80%. The CVI involves the mean I-CVI. In studies with six or more judges, an I-CVI no lower than 0.78 is recommended^(11,12)^.

## RESULTS

Throughout the process of cultural adaptation, the original version of the instrument underwent several modifications, represented in six different versions, as outlined in Chart 1. The results of each study stage during the translation and cultural adaptation are described in detail below.

**Chart 1:** Versions of the instrument

**Versão original**	**Primeira versão (obtida após a síntese das traduções)**	**Segunda versão (obtida após a análise dos especialistas)**	**Terceira versão (obtida após a análise da população-alvo)**	**Quarta versão (obtida por meio da retrotradução)**	**Quinta versão (obtida após a análise da retrotradução)**	**Versão final (obtida após o estudo piloto)**
My child communicates by using (check all that apply):	Minha criança comunica-se usando (assinale todas as opções aplicáveis):	Meu/Minha filho (a) comunica-se usando (assinale todas as opções aplicáveis):	Meu/Minha filho (a) comunica-se usando (assinale todas as opções aplicáveis):	My child communicates through (check all applicable options):	Meu/Minha filho (a) comunica-se usando (assinale todas as opções aplicáveis):	Meu/Minha filho (a) comunica-se usando (assinale todas as opções aplicáveis):
Speech Pictures/Photos	() Fala; ()Imagens/Fotos; () Sistema de comunicação de alta tecnologia (tecnologia assistiva); () Língua de sinais; () Placas de comunicação (Comunicação aumentativa/alternativa); () Outro:	() Fala; () Imagens/Fotos; () Sistema de comunicação de alta tecnologia (tecnologia assistiva); () Língua de sinais; () Prancha de comunicação (Comunicação aumentativa/alternativa); () Outro:	() Fala;	() Speech;	() Fala;	() Fala;
High Tech Communication System Sign Language Communication Board			() Imagens/Fotos;	() Images/Photos;	() Imagens/Fotos;	() Imagens/Fotos;
Other:			() Sistema de comunicação de alta tecnologia (tecnologia assistiva);	() High-technology communication systems (assistive technology);	() Sistema de comunicação de alta tecnologia (tecnologia assistiva);	() Sistema de comunicação de alta tecnologia (tecnologia assistiva);
			() Língua de sinais;	() Sign language;	() Língua de sinais;	() Língua de sinais;
			() Prancha de comunicação (Comunicação aumentativa/alternativa);	() Communication boards (augmentative/alternative communication);	() Prancha de comunicação (Comunicação aumentativa/alternativa);	() Prancha de comunicação (Comunicação aumentativa/alternativa);
			() Gestos;	() Gestures	() Gestos;	() Gestos;
			() Outro:	() Other:	() Outro:	() Outro: ____________________________
My child began to speak at about 2 years 3 years 4 years 5 years after 5 years	Meu filho começou a falar por volta dos: () 2 anos () 3 anos () 4 anos ()5 anos ()depois dos 5 anos.	Meu/minha filho (a) começou a falar por volta dos: () 2 anos () 3 anos () 4 anos ()5 anos ()depois dos 5 anos.	Meu/minha filho (a) começou a falar por volta dos: () 1 ano () 2 anos () 3 anos () 4 anos ()5 anos () depois dos 5 anos, () não fala	My child began speaking at around:	Meu/minha filho (a) começou a falar por volta dos: () 1 ano () 2 anos () 3 anos () 4 anos ()5 anos () depois dos 5 anos, () não fala	Meu/minha filho (a) começou a falar por volta dos:
				() 1 year old () 2 years old () 3 years old () 4 years old () 5 years old () after 5 years old () he/she does not speak		() 1 ano () 2 anos () 3 anos () 4 anos () 5 anos () depois dos 5 anos () não fala
On a scale of 1	Em uma escala de 1 à	Em uma escala de 1 a 10, na qual 1 é incompreensível	Em uma escala de 0 a 10, na qual 0 é ausência de fala e 10 é fala completamente compreensível, como você classificaria a fala do seu/sua filho(a)?	On a scale from 0 to 10, in which 0 is the absence of speech and 10 fully intelligible speech, how would you classify your child’s speech?	Em uma escala de 0 a 10, na qual 0 é ausência de fala e 10 é fala completamente compreensível, como você classificaria a fala do seu/sua filho(a)?	Em uma escala de 0 a 10, na qual 0 é ausência de fala e 10 é fala completamente compreensível, como você classificaria a fala do seu/sua filho(a)?
to 10, where 1 is completely unintelligible and	10, na qual 1 é completamente incompreensível e	e 10 é completamente compreensível, como				() 0 () 1 () 2 () 3 () 4 () 5 () 6 () 7 () 8 () 9 () 10
10 is completely	10 é completamente compreensível, como	você classificaria a fala				
intelligible, how	você classificaria a fala	do seu filho(a)?				
would you rate your child's speech?	do seu filho?					
Have you been told that your child has oral motor difficulties? Yes No	Já lhe disseram que seu filho tem dificuldades motoras orais?	Já lhe disseram que seu filho(a) tem dificuldades motoras orais?	Já lhe disseram que seu/sua filho(a) tem dificuldades motoras orais?	Have you ever been told that your child has oral motor difficulties?	Já lhe disseram que seu/sua filho(a) tem dificuldades motoras orais?	Já lhe disseram que seu/sua filho(a) tem dificuldades motoras orais?
		() sim () não	() sim () não	() yes () no	() sim () não	() sim () não
Have you been told that your child has apraxia or dyspraxia? Yes No	Já lhe disseram que seu filho tem apraxia ou dispraxia? () sim () não	Já lhe disseram que seu filho/filha tem apraxia ou dispraxia? () sim () não	Já lhe disseram que seu/sua filho(a) tem apraxia de fala?	Have you ever been told that your child has speech apraxia?	Já lhe disseram que seu/sua filho(a) tem apraxia de fala?	Já lhe disseram que seu/sua filho(a) tem apraxia de fala?
			() sim () não	() yes () no	() sim () não	() sim () não
People who know my child well have difficulty understanding his/her speech	Pessoas próximas ao meu filho têm dificuldade em entender a sua fala	Pessoas que já conhecem o meu/minha filho(a) têm dificuldade em entender a sua fala	Pessoas que já conhecem o meu/minha filho(a) têm dificuldade em entender a sua fala: () sempre () frequentemente () às vezes () nunca	People who already know my child have difficulties understanding his/her speech:	Pessoas que já conhecem o meu/minha filho(a) têm dificuldade em entender a sua fala: () sempre () frequentemente () às vezes () nunca	Pessoas que já conhecem o meu/minha filho(a) têm dificuldade em entender a sua fala:
				() always () often () sometimes () never		() Sempre () Frequentemente
						() Às vezes () Nunca
People who first meet my child have difficulty understanding his/her speech	Pessoas que conhecem o meu filho pela primeira vez têm dificuldade em entender a sua fala	Pessoas que conhecem o meu/minha filho (a) pela primeira vez têm dificuldade em entender a sua fala	Pessoas que conhecem o meu/minha filho (a) pela primeira vez têm dificuldade em entender a sua fala:	People who meet my child for the first time have difficulties understanding his/her speech:	Pessoas que conhecem o meu/minha filho (a) pela primeira vez têm dificuldade em entender a sua fala:	Pessoas que conhecem o meu/minha filho (a) pela primeira vez têm dificuldade em entender a sua fala:
			() sempre () frequentemente () às vezes () nunca	() always () often () sometimes () never	() sempre () frequentemente () às vezes () nunca	() Sempre () Frequentemente
						() Às vezes () Nunca
My child communicates primarily by using speech	Meu filho se comunica principalmente através da fala	Meu filho(a) se comunica principalmente através da fala	Meu/Minha filho(a) se comunica principalmente através da fala:	My child communicates mainly through speech:	Meu/Minha filho(a) se comunica principalmente através da fala:	Meu/Minha filho(a) se comunica principalmente através da fala:
			() sempre () frequentemente () às vezes () nunca	() always () often () sometimes () never	() sempre () frequentemente () às vezes () nunca	() Sempre () Frequentemente
						() Às vezes () Nunca
When someone can't understand my child's speech, family members interpret for him or her	Quando alguém não consegue entender a fala do meu filho, os membros da família a interpretam	Quando alguém não consegue entender a fala do(a) meu/minha filho(a), os membros da família a interpretam	Quando alguém não consegue entender a fala do(a) meu/minha filho(a), os membros da família a interpretam:	When someone does not understand my child’s speech, family members interpret it:	Quando alguém não consegue entender a fala do(a) meu/minha filho(a), os membros da família a interpretam:	Quando alguém não consegue entender a fala do(a) meu/minha filho(a), os membros da família a interpretam:
			() sempre () frequentemente () às vezes () nunca	() always () often () sometimes () never	() sempre () frequentemente () às vezes () nunca	() Sempre () Frequentemente
						() Às vezes () Nunca
In infancy, my child made cooing sounds (single sounds)	Na infância, o meu filho fazia sons murmurando (sons únicos)	Na infância, meu/minha filho(a) produzia sons isolados	Na infância, meu/minha filho(a) produzia sons isolados:	During childhood, my child produced isolated sounds:	Na infância, meu/minha filho(a) produzia sons isolados:	Na infância, meu/minha filho(a) produzia sons isolados (sons individuais, por exemplo: sons de consoantes ou de vogais):
			() sempre () frequentemente () às vezes () nunca	() always () often () sometimes () never	() sempre () frequentemente () às vezes () nunca	() Sempre () Frequentemente
						() Às vezes () Nunca
In infancy, my child babbled strings of sounds	Na infância, o meu filho balbuciava sequências de sons	Na infância, o meu/minha filho(a) balbuciava sequências de sons	Na infância, o meu/minha filho(a) balbuciava sequências de sons:	During childhood, my child babbled sound sequences:	Na infância, o meu/minha filho(a) balbuciava sequências de sons:	Na infância, o meu/minha filho(a) balbuciava sequências de sons (geralmente associando consoante + vogal, ex: ma ma):
			() sempre () frequentemente () às vezes () nunca	() always () often () sometimes () never	() sempre () frequentemente () às vezes () nunca	() Sempre () Frequentemente
						() Às vezes () Nunca
My child had difficulty sucking and swallowing liquids in infancy	Na infância, o meu filho tinha dificuldade em sugar e engolir líquidos	Na infância, meu/minha filho(a) tinha dificuldade em sugar e engolir líquidos	Na infância, meu/minha filho(a) tinha dificuldade em sugar e engolir líquidos:	During childhood, my child had difficulties sucking and swallowing liquids:	Na infância, meu/minha filho(a) tinha dificuldade em sugar e engolir líquidos:	Na infância, meu/minha filho(a) tinha dificuldade em sugar e engolir líquidos:
			() sempre () frequentemente () às vezes () nunca	() always () often () sometimes () never	() sempre () frequentemente () às vezes () nunca	() Sempre () Frequentemente
						() Às vezes () Nunca
My child had feeding difficulties when s/he started eating solid foods	Meu filho teve dificuldades de alimentação quando começou a comer alimentos sólidos	Meu/minha filho(a) teve dificuldades de alimentação quando começou a comer alimentos sólidos	Meu/minha filho(a) teve dificuldades de alimentação quando começou a comer alimentos sólidos:	My child had difficulties eating when he/she started having solid foods:	Meu/minha filho(a) teve dificuldades de alimentação quando começou a comer alimentos sólidos:	Meu/minha filho(a) teve dificuldades de alimentação quando começou a comer alimentos sólidos:
			() sempre () frequentemente () às vezes () nunca	() always () often () sometimes () never	() sempre () frequentemente () às vezes () nunca	() Sempre () Frequentemente
						() Às vezes () Nunca
My child currently has difficulties with swallowing liquids	Meu filho atualmente tem dificuldades em engolir líquidos.	Meu/minha filho(a) atualmente tem dificuldades em engolir líquidos.	Meu/minha filho(a) atualmente tem dificuldades em engolir líquidos:	My child currently has difficulties swallowing liquids:	Meu/minha filho(a) atualmente tem dificuldades em engolir líquidos:	Meu/minha filho(a) atualmente tem dificuldades em engolir líquidos:
			() sempre () frequentemente () às vezes () nunca	() always () often () sometimes () never	() sempre () frequentemente () às vezes () nunca	() Sempre () Frequentemente
						() Às vezes () Nunca
My child currently has difficulties with feeding/eating	Meu filho atualmente tem dificuldades em se alimentar/comer	Meu/minha filho(a) atualmente tem dificuldades em comer	Meu/minha filho(a) atualmente tem dificuldades em comer	My child currently has difficulties eating:	Meu/minha filho(a) atualmente tem dificuldades em comer	Meu/minha filho(a) atualmente tem dificuldades em comer:
			() sempre () frequentemente () às vezes () nunca	() always () often () sometimes () never	() sempre () frequentemente () às vezes () nunca	() Sempre () Frequentemente
						() Às vezes () Nunca
My child had low tone in the muscles of the face (lips, tongue, cheeks) in infancy	Na infância, meu filho tinha pouca força nos músculos do rosto (lábios, língua e bochechas)	Na infância, meu/minha filho(a) tinha pouca força nos músculos do rosto e da boca (lábios, língua e bochechas)	Na infância, meu/minha filho(a) tinha pouca força nos músculos do rosto e da boca (lábios, língua e bochechas):	During childhood, my child had little muscle strength on the face and in the mouth (lips, tongue, and cheeks):	Na infância, meu/minha filho(a) tinha pouca força nos músculos do rosto e da boca (lábios, língua e bochechas):	Na infância, meu/minha filho(a) tinha pouca força nos músculos do rosto e da boca (lábios, língua e bochechas):
			() sempre () frequentemente () às vezes () nunca	() always () often () sometimes () never	() sempre () frequentemente () às vezes () nunca	() Sempre () Frequentemente
						() Às vezes () Nunca
My child currently has low tone in the muscles of the face (lips, tongue, cheeks)	Meu filho atualmente tem baixa força nos músculos da face (lábios, língua, bochechas)	Meu/Minha filho(a) atualmente tem pouca força nos músculos do rosto e da boca (lábios, língua, bochechas)	Meu/Minha filho(a) atualmente tem pouca força nos músculos do rosto e da boca (lábios, língua, bochechas):	My child currently has little muscle strength on the face and in the mouth (lips, tongue, and cheeks):	Meu/Minha filho(a) atualmente tem pouca força nos músculos do rosto e da boca (lábios, língua, bochechas):	Meu/Minha filho(a) atualmente tem pouca força nos músculos do rosto e da boca (lábios, língua, bochechas):
			() sempre () frequentemente () às vezes () nunca	() always () often () sometimes () never	() sempre () frequentemente () às vezes () nunca	() Sempre () Frequentemente
						() Às vezes () Nunca
My child was late (delayed) in beginning to speak	Meu filho começou a falar tarde (atrasado).	Meu/minha filho(a) começou a falar tarde (atrasado).	Meu/minha filho(a) começou a falar tarde (atrasado):	My child began to talk late (delayed):	Meu/minha filho(a) começou a falar tarde (atrasado):	-
			() sempre () frequentemente () às vezes () nunca	() always () often () sometimes () never	() sempre () frequentemente () às vezes () nunca	
My child makes the same speech errors consistently	Meu filho comete consistentemente os mesmos erros de fala	Meu/minha filho(a) comete sempre os mesmos erros de fala	Meu/minha filho(a) comete os mesmos erros de fala:	My child makes the same speech errors:	Meu/minha filho(a) comete os mesmos erros de fala:	Meu/minha filho(a) comete os mesmos erros de fala (por exemplo, omite ou substitui os sons, de forma consistente, como o som de “s” por “t”):
			() sempre () frequentemente () às vezes () nunca	() always () often () sometimes () never	() sempre () frequentemente () às vezes () nunca	() Sempre () Frequentemente
						() Às vezes () Nunca
Sometimes, my child can say a word but at other times, my child has difficulty saying the same word	Às vezes, meu filho consegue falar uma palavra, mas em outras ocasiões, tem dificuldade em falar a mesma palavra	Às vezes, meu/minha filho(a) consegue falar uma palavra, mas em outras ocasiões, tem dificuldade em falar a mesma palavra.	Às vezes, meu/minha filho(a) consegue falar uma palavra, mas em outras ocasiões, tem dificuldade em falar a mesma palavra:	Sometimes, my child can say a word, while at other times he/she has difficulties saying that same word:	Às vezes, meu/minha filho(a) consegue falar uma palavra, mas em outras ocasiões, tem dificuldade em falar a mesma palavra:	Às vezes, meu/minha filho(a) consegue falar uma palavra, mas em outras ocasiões, tem dificuldade em falar a mesma palavra:
			() sempre () frequentemente () às vezes () nunca	() always () often () sometimes () never	() sempre () frequentemente () às vezes () nunca	() Sempre () Frequentemente
						() Às vezes () Nunca
My child is understandable when s/he says single words, but has greater difficulty in conversation	Meu filho é compreendido quando fala palavras isoladas, mas tem maior dificuldade em conversar	Meu/minha filho(a) é compreendido quando fala palavras isoladas, mas tem maior dificuldade em conversar	Meu/minha filho(a) é compreendido quando fala palavras isoladas, mas tem maior dificuldade em conversar:	My child is understood when he/she says isolated words but has greater difficulties in conversations:	Meu/minha filho(a) é compreendido quando fala palavras isoladas, mas tem maior dificuldade em conversar:	Meu/minha filho(a) é compreendido quando fala palavras isoladas, mas tem maior dificuldade em conversar:
			() sempre () frequentemente () às vezes () nunca	() always () often () sometimes () never	() sempre () frequentemente () às vezes () nunca	() Sempre () Frequentemente
						() Às vezes () Nunca
My child uses a few sounds, but does not make many different sounds	Meu filho usa alguns sons, mas não produz muitos sons diferentes	Meu/minha filho(a) usa alguns sons, mas não produz muitos sons diferentes	Meu/minha filho(a) fala alguns sons, mas não produz sons diferentes:	My child makes some sounds but does not produce different sounds:	Meu/minha filho(a) fala alguns sons, mas não produz sons diferentes:	Meu/minha filho(a) fala alguns sons, mas não produz sons diferentes:
			() sempre () frequentemente () às vezes () nunca	() always () often () sometimes () never	() sempre () frequentemente () às vezes () nunca	() Sempre () Frequentemente
						() Às vezes () Nunca
My child can sing the words in songs more clearly than s/he can say them when speaking	Meu filho consegue cantar as palavras das músicas com mais clareza do que quando as fala	Meu/minha filho(a) consegue cantar as palavras das músicas com mais clareza do que quando as fala.	Meu/minha filho(a) consegue cantar as palavras das músicas com mais clareza do que quando as fala:	My child can sing the lyrics of a song more clearly than when he/she speaks them:	Meu/minha filho(a) consegue cantar as palavras das músicas com mais clareza do que quando as fala:	Meu/minha filho(a) consegue cantar as palavras das músicas com mais clareza do que quando as fala:
			() sempre () frequentemente () às vezes () nunca	() always () often () sometimes () never	() sempre () frequentemente () às vezes () nunca	() Sempre () Frequentemente
						() Às vezes () Nunca
My child shows very slow improvement in speech therapy	Meu filho apresenta melhora muito lenta na terapia de fala	Meu/minha filho(a) apresenta melhora muito lenta na terapia fonoaudiológica	Meu/minha filho(a) apresenta melhora muito lenta na terapia fonoaudiológica:	My child improves very slowly in speech-language therapy:	Meu/minha filho(a) apresenta melhora muito lenta na terapia fonoaudiológica:	Meu/minha filho(a) apresenta melhora muito lenta na terapia fonoaudiológica:
			() sempre () frequentemente () às vezes () nunca	() always () often () sometimes () never	() sempre () frequentemente () às vezes () nunca	() Sempre () Frequentemente
						() Às vezes () Nunca
My child seems to be struggling so hard to say words and sounds	Meu filho parece se esforçar muito para produzir palavras e sons	Meu/minha filho(a) parece se esforçar muito para produzir palavras e sons	Meu/minha filho(a) parece se esforçar muito para produzir palavras e sons:	My child seems to make a great effort to produce words and sounds:	Meu/minha filho(a) parece se esforçar muito para produzir palavras e sons:	Meu/minha filho(a) parece se esforçar muito para produzir palavras e sons:
			() sempre () frequentemente () às vezes () nunca	() always () often () sometimes () never	() sempre () frequentemente () às vezes () nunca	() Sempre () Frequentemente
						() Às vezes () Nunca
My child speaks rapidly	Meu filho fala rapidamente	Meu/minha filho(a) fala rapidamente	Meu/minha filho(a) fala rapidamente: () sempre () frequentemente () às vezes () nunca	My child speaks hastily:	Meu/minha filho(a) fala rapidamente: () sempre () frequentemente () às vezes () nunca	Meu/minha filho(a) fala rapidamente:
				() always () often () sometimes () never		() Sempre () Frequentemente
						() Às vezes () Nunca
My child has fluency (stuttering-like) difficulties when speaking	Meu filho tem dificuldades de fluência (como gagueira) quando fala	Meu/minha filho(a) tem dificuldades de fluência (como gagueira) quando fala	Meu/minha filho(a) tem dificuldades de fluência (como gagueira) quando fala:	My child had fluency difficulties (such as stuttering) when he/she speaks:	Meu/minha filho(a) tem dificuldades de fluência (como gagueira) quando fala:	Meu/minha filho(a) tem dificuldades de fluência (como gagueira) quando fala:
			() sempre () frequentemente () às vezes () nunca	() always () often () sometimes () never	() sempre () frequentemente () às vezes () nunca	() Sempre () Frequentemente
						() Às vezes () Nunca
My child has difficulty hearing	Meu filho tem dificuldade de audição	Meu/minha filho(a) tem dificuldade de audição	Meu/minha filho(a) tem dificuldade de audição:	My child has hearing difficulties:	Meu/minha filho(a) tem dificuldade de audição:	Meu/minha filho(a) tem dificuldade de audição:
			() sempre () frequentemente () às vezes () nunca	() always () often () sometimes () never	() sempre () frequentemente () às vezes () nunca	() Sempre () Frequentemente
						() Às vezes () Nunca
My child has more difficulty saying longer words than shorter words	Meu filho tem mais dificuldade em falar palavras longas do que palavras curtas	Meu/minha filho(a) tem mais dificuldade em falar palavras longas do que palavras curtas	Meu/minha filho(a) tem mais dificuldade em falar palavras longas do que palavras curtas:	My child has greater difficulty speaking longer than shorter words:	Meu/minha filho(a) tem mais dificuldade em falar palavras longas do que palavras curtas:	Meu/minha filho(a) tem mais dificuldade em falar palavras longas do que palavras curtas:
			() sempre () frequentemente () às vezes () nunca	() always () often () sometimes () never	() sempre () frequentemente () às vezes () nunca	() Sempre () Frequentemente
						() Às vezes () Nunca
My child has more difficulty speaking when s/he is using longer phrases or sentences	Meu filho tem mais dificuldade em falar quando utiliza frases ou sentenças mais longas	Meu/minha filho(a) tem mais dificuldade em falar quando utiliza frases ou sentenças mais longas	Meu/minha filho(a) tem mais dificuldade em falar quando utiliza frases ou sentenças mais longas:	My child has greater difficulty speaking when he/she uses longer phrases or sentences:	Meu/minha filho(a) tem mais dificuldade em falar quando utiliza frases ou sentenças mais longas:	Meu/minha filho(a) tem mais dificuldade em falar quando utiliza frases ou sentenças mais longas:
			() sempre () frequentemente () às vezes () nunca	() always () often () sometimes () never	() sempre () frequentemente () às vezes () nunca	() Sempre () Frequentemente
						() Às vezes () Nunca
My child has difficulty saying some consonant sounds	Meu filho tem dificuldade em falar alguns sons de consoantes	Meu/minha filho(a) tem dificuldade em falar algumas consoantes	Meu/minha filho(a) tem dificuldade em falar algumas consoantes:	My child has difficulty saying some consonants:	Meu/minha filho(a) tem dificuldade em falar algumas consoantes:	Meu/minha filho(a) tem dificuldade em falar algumas consoantes:
			() sempre () frequentemente () às vezes () nunca	() always () often () sometimes () never	() sempre () frequentemente () às vezes () nunca	() Sempre () Frequentemente
						() Às vezes () Nunca
My child has difficulty saying some vowel sounds	Meu filho tem dificuldade em falar alguns sons de vogais	Meu/minha filho(a) tem dificuldade em falar algumas vogais	Meu/minha filho(a) tem dificuldade em falar algumas vogais:	My child has difficulty saying some vowels:	Meu/minha filho(a) tem dificuldade em falar algumas vogais:	Meu/minha filho(a) tem dificuldade em falar algumas vogais:
			() sempre () frequentemente () às vezes () nunca	() always () often () sometimes () never	() sempre () frequentemente () às vezes () nunca	() Sempre () Frequentemente
						() Às vezes () Nunca
My child often reverses sounds in words (e.g., aminal for animal)	Meu filho frequentemente inverte sons em palavras (por exemplo: aminal para animal)	Meu/minha filho(a) frequentemente inverte sons em palavras (por exemplo: aminal para animal)	Meu/minha filho(a) frequentemente inverte sons em palavras (por exemplo: aminal para animal):	My child often inverts sounds in words (for example, aminal for animal):	Meu/minha filho(a) frequentemente inverte sons em palavras (por exemplo: aminal para animal):	Meu/minha filho(a) inverte sons em palavras (por exemplo: aminal para animal):
			() sempre () frequentemente () às vezes () nunca	() always () often () sometimes () never	() sempre () frequentemente () às vezes () nunca	() Sempre () Frequentemente
						() Às vezes () Nunca
My child has difficulty with the rhythm of speech (speech sounds choppy, or sometimes slow and sometimes fast)	Meu filho tem dificuldade com o ritmo da fala (a fala parece variável, às vezes lenta e às vezes rápida)	Meu/minha filho(a) tem dificuldade com o ritmo da fala (a fala parece variável, às vezes lenta e às vezes rápida)	Meu/minha filho(a) tem dificuldade com o ritmo da fala (às vezes lenta e às vezes rápida):	My child has difficulties with speech rhythm (sometimes slow, sometimes fast):	Meu/minha filho(a) tem dificuldade com o ritmo da fala (às vezes lenta e às vezes rápida):	Meu/minha filho(a) tem dificuldade com o ritmo da fala (às vezes lenta e às vezes rápida):
			() sempre () frequentemente () às vezes () nunca	() always () often () sometimes () never	() sempre () frequentemente () às vezes () nunca	() Sempre () Frequentemente
						() Às vezes () Nunca
My child prolongs vowel sounds	Meu filho prolonga os sons das vogais	Meu/minha filho(a) prolonga os sons das vogais	Meu/minha filho(a) prolonga os sons das vogais:	My child prolongs vowel sounds:	Meu/minha filho(a) prolonga os sons das vogais:	Meu/minha filho(a) prolonga os sons das vogais:
			() sempre () frequentemente () às vezes () nunca	() always () often () sometimes () never	() sempre () frequentemente () às vezes () nunca	() Sempre () Frequentemente
						() Às vezes () Nunca
My child leaves out sounds in words	Meu filho omite sons nas palavras	Meu/minha filho(a) omite sons nas palavras	Meu/minha filho(a) omite sons nas palavras:	My child omits sounds in words:	Meu/minha filho(a) omite sons nas palavras:	Meu/minha filho(a) omite sons nas palavras:
			() sempre () frequentemente () às vezes () nunca	() always () often () sometimes () never	() sempre () frequentemente () às vezes () nunca	() Sempre () Frequentemente
						() Às vezes () Nunca
My child leaves out syllables in words	Meu filho omite sílabas nas palavras	Meu/minha filho(a) não fala algumas sílabas das palavras	Meu/minha filho(a) não fala algumas sílabas das palavras:	My child does not speak some syllables in words:	Meu/minha filho(a) não fala algumas sílabas das palavras:	Meu/minha filho(a) omite algumas sílabas das palavras:
			() sempre () frequentemente () às vezes () nunca	() always () often () sometimes () never	() sempre () frequentemente () às vezes () nunca	() Sempre () Frequentemente
						() Às vezes () Nunca
My child's speech sounds hypernasal (as if it's coming through his/her nose)	Meu filho fala sons hipernasais (como se estivessem saindo pelo nariz).	Meu/minha filho(a) fala sons hipernasais (como se estivessem saindo pelo nariz).	Meu/minha filho(a) fala sons hipernasais (como se estivessem saindo pelo nariz):	My child speaks with hypernasal sounds (as if coming through the nose):	Meu/minha filho(a) fala sons hipernasais (como se estivessem saindo pelo nariz):	Meu/minha filho(a) fala sons hipernasais (como se estivessem saindo pelo nariz):
			() sempre () frequentemente () às vezes () nunca	() always () often () sometimes () never	() sempre () frequentemente () às vezes () nunca	() Sempre () Frequentemente
						() Às vezes () Nunca
My child talks less with people outside of the circle of friends and family	Meu filho fala menos com pessoas de fora do círculo de amigos e família.	Meu/minha filho(a) fala menos com pessoas de fora do círculo de amigos e família.	Meu/minha filho(a) fala menos com pessoas de fora do círculo de amigos e família:	My child speaks less with people outside his/her circle of friends and family:	Meu/minha filho(a) fala menos com pessoas de fora do círculo de amigos e família:	Meu/minha filho(a) fala menos com pessoas de fora do círculo de amigos e família:
			() sempre () frequentemente () às vezes () nunca	() always () often () sometimes () never	() sempre () frequentemente () às vezes () nunca	() Sempre () Frequentemente
						() Às vezes () Nunca
It is hard for my child to imitate a word that I say	É difícil para meu filho imitar uma palavra que eu falo	É difícil para meu/minha filho(a) imitar uma palavra que eu falo	É difícil para meu/minha filho(a) imitar uma palavra que eu falo:	It is difficult for my child to imitate a word I speak:	É difícil para meu/minha filho(a) imitar uma palavra que eu falo:	É difícil para meu/minha filho(a) imitar uma palavra que eu falo:
			() sempre () frequentemente () às vezes () nunca	() always () often () sometimes () never	() sempre () frequentemente () às vezes () nunca	() Sempre () Frequentemente
						() Às vezes () Nunca
My child's speech is easier to understand when s/he is saying familiar words	A fala do meu filho é mais fácil de entender quando ele está dizendo palavras familiares	A fala do meu/minha filho(a) é mais fácil de entender quando ele(a) está dizendo palavras familiares	A fala do meu/minha filho(a) é mais fácil de entender quando ele(a) está dizendo palavras familiares:	It is easier to understand my child’s speech when he/she is saying familiar words:	A fala do meu/minha filho(a) é mais fácil de entender quando ele(a) está dizendo palavras familiares:	A fala do meu/minha filho(a) é mais fácil de entender quando ele(a) está dizendo palavras familiares:
			() sempre () frequentemente () às vezes () nunca	() always () often () sometimes () never	() sempre () frequentemente () às vezes () nunca	() Sempre () Frequentemente
						() Às vezes () Nunca
My child understands more than s/he can say	Meu filho compreende mais do que ele pode falar	Meu/minha filho(a) compreende mais do que ele(a) pode falar	Meu/minha filho(a) compreende mais do que ele(a) pode falar:	My child understands more than he/she can speak:	Meu/minha filho(a) compreende mais do que ele(a) pode falar:	Meu/minha filho(a) compreende mais do que ele(a) pode falar:
			() sempre () frequentemente () às vezes () nunca	() always () often () sometimes () never	() sempre () frequentemente () às vezes () nunca	() Sempre () Frequentemente
						() Às vezes () Nunca
My child may unexpectedly say a word or phrase perfectly, but then s/he can't repeat it	Meu filho pode espontaneamente falar uma palavra ou frase perfeitamente, mas ele não consegue repeti-la	Meu/minha filho(a) pode espontaneamente falar uma palavra ou frase perfeitamente, mas ele(a) não consegue repeti-la	Meu/minha filho(a) pode espontaneamente falar uma palavra ou frase, mas ele(a) não consegue repeti-la:	My child can spontaneously say a word or phrase, but he/she cannot repeat it:	Meu/minha filho(a) pode inesperadamente falar uma palavra ou frase, mas ele(a) não consegue repeti-la:	Meu/minha filho(a) pode inesperadamente falar uma palavra ou frase, mas ele(a) não consegue repeti-la:
			() sempre () frequentemente () às vezes () nunca	() always () often () sometimes () never	() sempre () frequentemente () às vezes () nunca	() Sempre () Frequentemente
						() Às vezes () Nunca
My child has difficulty with grammar	Meu filho tem dificuldade com a gramática	Meu/minha filho(a) tem dificuldade com a gramática	Meu/minha filho(a) tem dificuldade com a gramática:	My child has difficulties with grammar:	Meu/minha filho(a) tem dificuldade com a gramática:	Meu/minha filho(a) tem dificuldade com a gramática:
			() sempre () frequentemente () às vezes () nunca	() always () often () sometimes () never	() sempre () frequentemente () às vezes () nunca	() Sempre () Frequentemente
						() Às vezes () Nunca
My child is frustrated when people don't understand what s/he is saying	Meu filho fica frustrado quando as pessoas não entendem o que ele está falando	Meu/minha filho(a) fica frustrado(a) quando as pessoas não entendem o que ele(a) está falando	Meu/minha filho(a) fica frustrado(a) quando as pessoas não entendem o que ele(a) está falando:	My child gets frustrated when people do not understand what he/she is saying:	Meu/minha filho(a) fica frustrado(a) quando as pessoas não entendem o que ele(a) está falando:	Meu/minha filho(a) fica frustrado(a) quando as pessoas não entendem o que ele(a) está falando:
			() sempre () frequentemente () às vezes () nunca	() always () often () sometimes () never	() sempre () frequentemente () às vezes () nunca	() Sempre () Frequentemente
						() Às vezes () Nunca

Source: The authors

### Stages with translation and synthesis of translations

At the end of the translation stage, it was observed that one version had a more literal translation of the items, while the other had more significant modifications. During the synthesis of the translations, there was considerable consistency between the two translations, differing primarily in the degree of literal correspondence to the original version. The synthesis was conducted by considering the version of each item that was more adapted to the context, resulting in a combination of both translations, which represented the first version of the instrument.

### Stage with the judges’ analysis of the synthesis

Most judges in this stage evaluated almost all items (97.77%) in the synthesis of the translations as very relevant, very viable, and appropriate. The results indicated a CVI of 0.97 for relevance and viability and 0.82 for appropriateness. Most judges evaluated only one item as inadequate due to the terms used (“murmuring sounds/unique sounds”), which were classified as difficult for parents to understand, thus requiring an adaptation to make them more comprehensible.

Also considering the judges' analysis, one aspect that needed adjustments was the differentiation of the child's sex (male/female). It was initially decided to use only the masculine gender in the synthesis to simplify the wording of the items, thereby avoiding the need for gender-specific agreement in each item. However, the judges felt that this approach made it more difficult for parents to identify with the instrument and diminished the sense of belonging that comes with the description of their child's sex. As a result, sex differentiation was used in all items.

It is important to clarify that each suggestion from the judges was analyzed by the research team, noting that even though some items presented an I-CVI lower than 80%, some of the considerations did not specifically refer to the content of the item. These considerations were related to general aspects, such as the differentiation of the participant’s child’s sex, which appeared in several items, and the replacement of the expression “my child.” Given the age range covered by the instrument, the participants' children could be 1 to 21 years old, and thus this expression might not be entirely appropriate for all age groups.

Additionally, it is worth noting that all the judges assessed the instructions and clarity of the instrument as appropriate, while the structure was judged as inadequate by only one of them. Therefore, after this stage, and based on consensus from the team responsible for conducting the study, the necessary modifications were made.

### Stage with the target-public’s analysis

This stage identified that the vast majority (32 out of the 44 items) were understood by all parents. Some aspects that caused difficulties and were pointed out as needing adaptation included the questions specifically referring to speech. The applicability of these questions was questioned when the child does not speak and communicates nonverbally.

There was also difficulty in understanding technical terms (such as oral motor difficulties, apraxia, dyspraxia, isolated sounds, and speech errors), with requests for definitions or examples. Additionally, participants asked for clarification regarding what would be considered as speech and what is meant by childhood. All participants judged the instructions, clarity, and structure of the instrument as appropriate.

Thus, the research team held a consensus meeting, and the necessary modifications were made. It is worth noting that, as in the previous stage, some changes were made even though they were not specifically pointed out by the participants. These modifications were identified as necessary by the team based on the analyses conducted collectively.

### Stage with back translation

After making the modifications based on the target audience’s analysis, a new version of the instrument was obtained, which was then subjected to back translation from Portuguese into English.

In the comparative analysis between the back translated and the original version, some differences were identified regarding verb tenses and the use of comparative degrees in certain items.

Additionally, a distinction was noted between the items “People who know my child well have difficulty understanding his/her speech” and “People who already know my child have difficulties understanding his/her speech,” which differ in intensity due to the use of the adverb “well.” The Portuguese version used the expression “Pessoas que já conhecem o meu/minha filho(a) têm dificuldade em entender a sua fala” (“People who already know my child have difficulties understanding his/her speech”). This was maintained because it represented the contrast with the expression in the following item: “Pessoas que conhecem o meu/minha filho(a) pela primeira vez têm dificuldade em entender a sua fala” (“People who meet my child for the first time have difficulty understanding what he/she says”).

Additionally, a difference was observed between the expressions “My child makes the same speech errors consistently” and “My child makes the same speech errors.” The adapted version maintained “Meu/minha filho(a) comete os mesmos erros de fala” (“My child makes the same speech errors”). In this item, it was considered that the adverb of manner (consistently) was already conveyed by the option “sempre” (always) in the response scale, thus representing individuals who consistently make the same speech errors.

Finally, a need for change was identified when comparing the sentences “My child may unexpectedly say a word or phrase perfectly, but then she/he can't repeat it” and “My child can spontaneously say a word or phrase, but he/she cannot repeat it,” as the terms “unexpectedly” and “spontaneously” do not convey the same meaning. Therefore, the following modification was made: the term “espontaneamente” (spontaneously) was replaced with “inesperadamente” (unexpectedly) in the sentence “Meu/minha filho(a) pode espontaneamente falar uma palavra ou frase, mas ele(a) não consegue repeti-la.”

### Stage with the pilot study

The age of the participants’ children with T21 ranged from 1.33 to 21 years, with a mean age of 9.33 years. Several suggestions were identified, including the need for clearer wording in some questions, difficulty in understanding the expression “força nos músculos da face” (strength in the facial muscles) and the term “apraxia de fala” (speech apraxia), responses that did not align with the questions, modification of the response scale to “yes” or “no,” reduction in the number of items, questions not applicable to parents of babies, questions with the same meaning, and the need to specify the age referred to by the term “na infância” (in childhood).

Most participants (64.28%) did not have difficulty understanding the meaning of the items, and none of the participants reported difficulty in completing the questionnaire online.

In the parents’ suggestions, it is important to clarify that they were presented individually by the participants, without recurrence, indicating that the aspects raised were not frequent and may not significantly impact comprehension. However, these aspects collectively led to difficulties, as evidenced by the percentage of parents who reported comprehension issues, even though the sample size was small.

Hence, the instrument was reviewed regarding clarity, and it was deemed appropriate to add examples of isolated sounds, sound sequences, and speech errors.

It is understood that the respondents had difficulties using the study’s response scale, given the indications of responses that do not align with the question, the need to modify the response scale to “yes” or “no,” and the reduction in the number of items. This is because analyzing the scale became more challenging, as it involved a gradation of the frequency of occurrence of characteristics rather than a yes/no choice.

However, the decision was made to maintain the scale as it allows for a more detailed understanding of the construct assessed by the instrument, as well as enabling comparability with other studies that have already used or will use the instrument in its original and adapted versions.

Regarding the suggestion that some questions had the same meaning, a proximity was identified between the items “My child began to speak around: () 1 year () 2 years () 3 years () 4 years () 5 years () after 5 years, () does not speak” and “My child started speaking late (delayed): () always () frequently () sometimes () never,” as the first question already captures the content addressed in the second.

Moreover, it would not necessarily be up to the parents to determine if their child's speech onset was delayed, even though they may do so intuitively by comparing their child to other children of similar age or with the same clinical condition, or if such a characteristic was indicated by a professional. Additionally, the scale presented in the second item is not suitable, as the response should be yes or no. Therefore, we decided to remove the second item.

As for the other items and questions, we believe that they refer to specific nuances of speech that may appear similar to a layperson but represent important analyses for the speech-language-hearing pathologist. These nuances are valuable and should be included in the instrument for a comprehensive evaluation.

Finally, regarding the suggestion to specify the age referred to by the term “in childhood,” this comment had already been raised during the analysis by both the experts and the target population. Therefore, it was decided to define the period in the preliminary instructions of the instrument, considering the limits established by the World Health Organization and adopted by the Brazilian Ministry of Health in the National Policy for Comprehensive Child Health Care, which defines childhood as the period from 0 to 9 years old^(13)^.

Additionally, the age limit (18 years) was defined for children with T21, whose characteristics will be investigated using the instrument. This establishes an equivalence with the end of adolescence and the beginning of adulthood, as outlined in the Statute of Children and Adolescents^(14)^.

Thus, the necessary modifications were made based on the consensus reached among the researchers who conducted this study, resulting in the final version of the instrument.

## DISCUSSION

The translation and cross-cultural adaptation carried out in this study was based on the methodology proposed by Borsa, Damásio, and Bandeira^(9)^, following the Guidelines of Beaton et al.^(15)^ and the International Test Commission (ITC)^(16)^. The entire process identified various methods for instrument translation and adaptation studies, as previously evidenced in a review in the field^(17)^.

Another literature review identified the variety of methods used in speech-language-hearing instrument translation and adaptation studies in Brazilian Portuguese, based on standardized instructions and specific guidelines^(18)^.

This aspect hinders the comparability between studies and the standardization of procedures for achieving the best results concerning the equivalence between the original and adapted versions. Therefore, there is a need to make efforts to reach a consensus among research groups in the field, aiming to systematize the evidence and standardize a methodological design to be followed in clinical instrument translation and adaptation studies.

In this perspective, it is important to highlight the scarcity of practical references regarding the procedures and analyses involved in the construction and adaptation of clinical instruments, an aspect still observed today^(19)^. Although there have been advancements in the field, especially in the context of psychometrics (in the development and adaptation of psychological instruments), with efforts to establish a consensus to guide researchers, there is still considerable methodological diversity and a lack of evidence to favor one approach over the others.

Thus, translation and adaptation are sometimes based on the opinions of experts and the procedures they recommend, considering their practical experience in the field and knowledge of international guidelines. This is a rich speech-language-hearing field to be explored, aiming to analyze evidence in the area, provide clinicians with tools, and promote cross-cultural studies and comparisons.

Thus, we will present the following considerations regarding the translation and adaptation process developed in this study, comparing its procedures with those recommended by international guidelines and researchers in the field.

### Stages with translation and synthesis of translations

Beaton et al.^(15)^ recommend that the translation process be carried out by two independent bilingual translators, whose native language is the target language, one of whom is knowledgeable about the concepts being examined and the other is not (naive translator). These authors also suggest that the translations should be synthesized through a consensus among the translators and an observer, resulting in a common translation^(15)^.

The ITC Guidelines^(16)^ recommend choosing translators, if possible, with experience in the content of the test and knowledge of evaluation principles, in addition to being native speakers of the language and living within the culture. Furthermore, they suggest a dual translation and reconciliation procedure, aimed at addressing the deficiencies and risks of relying on idiosyncrasies of simplified translations. In this approach, a third independent translator or a group of experts identifies and resolves any discrepancies between alternative translations and reconciles them into a single version^(16)^.

These recommendations were partially followed in the present study, in which the two translators were native Portuguese speakers and fluent in English. Both translators knew the topic of childhood apraxia of speech, as they were part of the research team, had publications in the field, and were familiar with the construct being evaluated. Additionally, the translations were synthesized through a consensus involving the two translators and the other two researchers participating in the study. Subsequently, the two translations and their synthesis were submitted to the judgment of expert judges.

This approach – selecting translators familiar with the topic, having the research team analyze the translations, making decisions jointly, and choosing the most appropriate content for the translation synthesis – proved to be very satisfactory. Additionally, the judgment made by the expert judges regarding the adequacy of the translation synthesis, based on access to the original version and the two translations, allowed for an external analysis by specialists in the evaluated construct, establishing equivalence between the original and translated versions, as explained below.

### Stage with the experts’ judgment

Beaton et al.^(15)^ argue that the role of the expert committee is to consolidate all versions of the questionnaire and develop what would be considered the pre-final version of the instrument for field testing. Hence, the committee is responsible for reviewing all translations and reaching a consensus on any discrepancies.

The ITC Guidelines^(16)^ define an “expert” as an individual or group with sufficient knowledge of (1) the languages involved, (2) the cultures, (3) the test content, and (4) the general principles of testing. From this perspective, they recommend compiling review data from evaluators to maximize the appropriateness of the test adaptation for target populations. Furthermore, they advocate for the use of native reviewers from the local culture and language to assess the test’s translation/adaptation.

These aspects were considered in the present study, as the expert judges possessed the necessary qualifications, being native speakers of the target language and culture, with knowledge of the test's content. They analyzed the two versions from the translation phase and evaluated each item in the synthesis of translations, assessing its adequacy, feasibility, and relevance, and making suggestions for modifications.

Based on this, the necessary adjustments were made through a consensus reached by the research team, which consolidated the modifications individually suggested by the judges and selected those deemed relevant. This methodology was chosen as it was more feasible within the study's context, considering that a consensus among all 10 judges who voluntarily participated in the research would not have been practical. This method successfully met the study's objectives, ensuring the appropriateness, feasibility, and adequacy of the expressions in the items, and their equivalence with the original version.

### Stage with the target-audience’s analysis

This stage is rarely explored in the existing literature in the field, yet it is important for assessing whether the items, instructions, and response scale are understandable to the target audience^(9)^.

In the present study, this stage was conducted through the analysis of a sample from the target population, proving to be crucial to adapt the instrument, with several pertinent and relevant contributions to its alignment with the Brazilian cultural context. Thus, it proved highly valuable for assessing the instrument's feasibility and the degree of understanding of the items' content. This step helped implement necessary adjustments prior to the back translation process (which would compare the translated and adapted version with the original version), making it indispensable at this stage of the study.

### Stage with back translation

Back translation is a type of validity check, highlighting major inconsistencies or conceptual errors in the translation. The translators should be unfamiliar with the concepts explored in the instrument^(15)^.

One disadvantage of back translation is that, if implemented in its most restrictive form, it will not allow for any revision of the target language version^(16)^. Therefore, the study must consider existing adaptations, which result in differences from the original version due to the new cultural context. In this regard, the use of multiple translation designs is recommended, such as a back translation to verify the target version originated from double translation and reconciliation by a group of experts^(16)^.

The present study followed these guidelines. The back translation was performed by an independent translator who had no access to either the original version or the translated and adapted target language version. The back translated version was compared with the original version by the research team, which identified existing inconsistencies, with emphasis on the adaptations made for the new cultural context and the preservation of the instrument's originally intended meaning to ensure that the content of the items was maintained.

The goal of this stage is to assess the extent to which the translated version reflects the content of the item, as proposed by the original version. It can be used as a practical tool for the researcher adapting the instrument to communicate with the author of the original instrument^(9)^. In this regard, it is recommended that the authors of the original version provide feedback on the back translated version^(20)^.

### Stage with the pilot study

Local studies can be used to evaluate the test, and the pilot study is a strategy to provide evidence that the test instructions and item content have a similar meaning for all target populations^(16)^. In this regard, it is relevant to administer a small-scale test of the adapted version of the instrument, employing not only the administration of the test and data analysis but, more importantly, conducting interviews with both the administrators and examinees to gather their feedback on the test itself^(16)^.

Ideally, 30 to 40 people should be tested, as this is the final stage of the adaptation process, testing the pre-final version on subjects or patients from the target population^(15)^. Hence, a limitation of the present study was the few participants in the pilot study, as only 14 individuals took part. However, based on the feedback provided by the volunteers, it was possible to observe and reflect on previously unrecognized aspects, such as the possibility of combining items from the instrument and modifying the response scale. Additionally, it allowed for the review of previously mentioned aspects, making the necessary adjustments and obtaining the final version of the translated and adapted instrument.

## CONCLUSION

The translation and cross-cultural adaptation verified content-based validity evidence for the Brazilian version of the Down Syndrome Speech Intelligibility Survey. This instrument is relevant for screening speech apraxia associated with T21 in the Brazilian population, considering the correspondence between the characteristics listed in the tool and the findings from the existing literature in the field. However, to verify its other evidence of validity and reliability, it will be necessary to submit it to the subsequent stages of the validation process.

The challenges of differential diagnosis of speech disorders in T21 are even greater, with difficulty in distinguishing what is specific to the genetic condition, apraxia, or other speech and language disorders, due to the various interfering variables in this analysis.

It is believed that, through the investigation of the characteristics of speech intelligibility in this population, facilitated by the instrument translated and adapted in this study, it will be possible to screen these individuals and later evaluate them with specialized diagnosis, with a detailed analysis of various aspects related to expressive and receptive language functioning.

The effort dedicated to the differential diagnosis of this disorder and the detailed investigation of its etiological aspects are essential to develop increasingly targeted interventions based on the existing deficits, in accordance with the clinical condition. This highlights the need for further research in the field to enhance both clinical and educational care.
