# Supplementary material for: Translation and cross-cultural adaptation of the Brazilian version of the Down Syndrome Speech Intelligibility Survey
Source: Codas. 2025 Mar 31;37(3):e20240079. doi: 10.1590/2317-1782/e20240079en (PMC11963884; doi:10.1590/2317-1782/e20240079en)
Supplement: Translation

# Translation: Tradução e adaptação transcultural da versão brasileira da *Down Syndrome Speech Intelligibility Survey*

**DOI:** 10.1590/2317-1782/e20240079pt

**Authors:** Julyane Feitoza Coêlho, Gustavo Lopez Estivalet, Francisco Tiago Meireles da Silva, Isabelle Cahino Delgado, Leandro de Araújo Pernambuco, Giorvan Ânderson dos Santos Alves

**Affiliations:** 1 Programa de Pós-graduação em Linguística, Universidade Federal da Paraíba – UFPB - João Pessoa (PB), Brasil.; 2 Departamento de Letras Estrangeiras Modernas, Universidade Federal da Paraíba – UFPB - João Pessoa (PB), Brasil.; 3 Departamento de Fonoaudiologia, Universidade Federal da Paraíba – UFPB - João Pessoa (PB), Brasil.; 4 Departamento de Fonoaudiologia, Universidade Federal de Pernambuco – UFPE - Recife (PE), Brasil.

**Keywords:** Síndrome de Down, Apraxias, Programas de Rastreamento, Diagnóstico, Diagnóstico Diferencial

## Abstract

**Objetivo:**

traduzir e adaptar transculturalmente para o português brasileiro o questionário “Down Syndrome Speech Intelligibility Survey”.

**Método:**

foram realizadas as etapas da adaptação transcultural: tradução do instrumento do idioma de origem para o idioma-alvo, síntese das versões traduzidas, avaliação da síntese por juízes experts, avaliação do instrumento pelo público-alvo, retrotradução e estudo-piloto.

**Resultados:**

após a tradução e síntese das traduções, na análise pelos juízes especialistas, a quase totalidade dos itens do instrumento (n=44, 97,77%) foi considerada muito relevante, muito viável e adequada pela maioria dos juízes. Na análise pela população-alvo, foram apontadas sugestões de mudança pelos pais, permitindo a realização de modificações para uma melhor compreensão do instrumento. A partir da retrotradução, foram identificadas algumas inconsistências da versão traduzida e adaptada em relação ao conteúdo proposto no instrumento original, sendo realizadas as devidas adequações. No estudo-piloto, foi identificada a necessidade de adaptar algumas perguntas; de excluir um dos itens, que apresentava conteúdo semelhante a outro; e de inserir orientações prévias para preenchimento, resultando na versão final do instrumento.

**Conclusão:**

A tradução e adaptação transcultural permitiu verificar as evidências de validade baseadas no conteúdo da versão brasileira do Down Syndrome Speech Intelligibility Survey.

## INTRODUÇÃO

A síndrome de Down é uma condição genética caracterizada por uma mudança na distribuição dos cromossomos nas células, apresentando, na maioria dos casos, um cromossomo extra no par 21^(1)^. Nesta perspectiva, atualmente há uma preferência pela utilização da denominação Trissomia do Cromossomo 21 (T21).

A T21 traz diversas repercussões no processo de desenvolvimento global, incluindo os prejuízos linguístico-comunicativos, estando esses déficits associados ao comprometimento cognitivo, motor, auditivo e ao desenvolvimento do sistema oromiofuncional. Considerando especificamente os atrasos e desafios relacionados à linguagem, ao longo do tempo, diversos prejuízos foram encontrados nos indivíduos com esta condição, particularmente no processamento fonológico e no desenvolvimento sintático. Neste contexto, estudo de revisão indicou que indivíduos com T21 podem apresentar desafios específicos com o processamento fonológico, devido aos déficits pronunciados na memória verbal de curto prazo^(2)^.

A fala de pessoas com T21 pode se encontrar alterada devido às características do sistema estomatognático desses indivíduos, bem como por dificuldades na programação de movimentos e na sequencialização necessárias à produção dos sons da fala. Nesses indivíduos, quando há um comprometimento na capacidade de programar voluntariamente os movimentos da fala, associado a diminuição na inteligibilidade de fala, inconsistência nos erros, dificuldades na sequencialização dos sons e movimentos orais, caracteriza-se clinicamente uma apraxia de fala^(3)^.

A Apraxia da Fala na Infância (AFI) é um transtorno dos sons da fala, de origem neurológica, que ocorre na infância, no qual a precisão e a consistência dos movimentos subjacentes à fala são prejudicadas na ausência de déficits neuromusculares. A AFI pode ocorrer como resultado de comprometimento neurológico conhecido; em associação com distúrbios neurocomportamentais complexos, de origem conhecida ou desconhecida; ou como um transtorno dos sons da fala neurogênico idiopático. O comprometimento central no planejamento e/ou programação de parâmetros espaço-temporais das sequências de movimentos resulta em erros na produção e na prosódia dos sons da fala^(4)^.

Essa condição foi identificada em indivíduos com T21, com uma prevalência de 11,1%, a partir da análise de amostras de fala de 45 participantes, com idades entre 10 e 20 anos, utilizando métodos e medidas perceptivas e acústicas do *Speech Disorders Classification System* (SDCS). Ainda, identificou-se que quase a totalidade da amostra de participantes com T21 apresentou algum tipo de transtorno dos sons da fala e de transtorno motor da fala^(5)^.

Nesse contexto, um dos desafios existentes reside no diagnóstico diferencial dos transtornos de fala, de modo que o processo de avaliação fonoaudiológica da linguagem na T21 envolve uma investigação em diversas etapas, que compreendem tanto o aspecto expressivo quanto o receptivo. Assim, a análise da linguagem abrange os diversos níveis linguísticos, podendo ainda envolver, em uma perspectiva multimodal, a análise da efetividade no uso de gestos e expressões faciais como auxílio à comunicação. Através disso, busca-se identificar os maiores prejuízos existentes e as potencialidades, para guiar a intervenção e estimular o desenvolvimento das habilidades linguístico-comunicativas.

A sobreposição de alterações distintas, aliada ao déficit intelectual característico da síndrome, demandam uma investigação pormenorizada e dificultam o diagnóstico diferencial dos transtornos de fala. Nesse contexto, outro desafio reside na carência de instrumentos disponíveis em português brasileiro e validados para serem aplicados com esse público.

Considerando a recomendação de submeter a população a testes específicos, de acordo com os aspectos epidemiológicos dos agravos a serem rastreados^(6)^, o rastreamento da AFI na T21 apresenta relevância. Sendo assim, o rastreamento permite identificar a ocorrência de características dessa condição nessa população específica, que apresenta uma prevalência considerável nestes indivíduos^(5)^. Nesse sentido, é de fundamental importância a utilização de instrumentos que permitam triar e indicar aqueles indivíduos que possuam sinais sugestivos de AFI e demandem uma avaliação direcionada para o transtorno.

Diante disso, o presente estudo busca contribuir para a investigação da apraxia de fala associada a essa condição genética, em indivíduos brasileiros com T21. Para isso, realizou-se a adaptação transcultural de um instrumento – *Down Syndrome Speech Intelligibility Survey* ^(7)^ – desenvolvido para aplicação junto aos pais de pessoas com T21, sendo considerado como uma ferramenta útil para o rastreamento desse transtorno motor da fala^(8)^.

A adaptação transcultural é um processo que permite produzir versões equivalentes de um instrumento traduzido para uma nova língua, o que favorece a utilização de instrumentos válidos já desenvolvidos e a realização de estudos transculturais. É desenvolvida por meio de metodologia própria, que envolve diversas etapas, como a tradução do instrumento do idioma de origem para o idioma-alvo, síntese das versões traduzidas, avaliação da síntese por juízes experts, avaliação do instrumento pelo público-alvo, tradução reversa e estudo-piloto^(9)^.

O referido instrumento foi escolhido para o processo de adaptação transcultural por ser o único disponível para a investigação dessas características especificamente nesse público. Cabe destacar que, este questionário possui grande relevância por ter sido utilizado para o primeiro levantamento amplamente realizado sobre as características da apraxia de fala na população com T21. O instrumento já foi traduzido e adaptado para o idioma turco, com resultados promissores, sendo identificado como uma ferramenta de triagem para um possível diagnóstico da apraxia de fala associada à T21^(8)^.

Ainda, a necessidade de adaptação desse instrumento se justifica por não existir nenhum questionário em língua portuguesa proposto para análise desses aspectos, cuja aplicação seja realizada com os pais de pessoas com T21, que se constituem em um importante público para a identificação dessas características de fala.

Assim, a adaptação transcultural desse questionário e sua posterior validação poderão contribuir na prática clínica dos fonoaudiólogos brasileiros, como um instrumento de rastreamento para auxiliar na identificação dos indivíduos que demandem uma avaliação específica para o diagnóstico diferencial das alterações de fala.

Diante do exposto, o objetivo do estudo é traduzir e adaptar transculturalmente o questionário “*Down Syndrome Speech Intelligibility Survey*” para o português brasileiro.

## MÉTODO

Todos os aspectos éticos foram considerados, em respeito à Resolução nº 466/12^(10)^. O projeto de pesquisa foi previamente submetido ao Comitê de Ética em Pesquisa com Seres Humanos de uma Instituição de Ensino Superior, sendo apreciado e aprovado por meio do parecer de número 5.127.264. Durante a coleta, o Termo de Consentimento Livre e Esclarecido foi apresentado ao participante, preservando o sigilo de todos os dados coletados. Ainda, foi obtida a autorização prévia da autora do instrumento original para a realização do processo de adaptação transcultural.

### Instrumento

O *Down Syndrome Speech Intelligibility Survey* ^(7)^ foi o instrumento traduzido e adaptado. Trata-se de um questionário composto por cinco perguntas e 40 sentenças, voltado para aplicação com pais de pessoas com T21. Nos itens que correspondem às sentenças, o informante deve graduar a frequência em que determinada característica está presente na fala de seu filho (sempre, frequentemente, às vezes ou nunca). A partir disso, os achados são interpretados qualitativamente pelo fonoaudiólogo.

### Procedimentos de coleta de dados

Esta pesquisa foi realizada durante o período de setembro de 2021 e agosto de 2023. O processo de adaptação transcultural foi conduzido conforme a metodologia proposta por Borsa, Damasio e Bandeira (2012), diante da sua viabilidade e aplicabilidade. Esse modelo metodológico considera seis etapas: (1) tradução do instrumento do idioma de origem para o idioma-alvo, (2) síntese das versões traduzidas, (3) avaliação da síntese por juízes experts, (4) avaliação do instrumento pelo público-alvo, (5) tradução reversa – retrotradução, e (6) estudo-piloto.

Inicialmente, foi realizada a tradução do instrumento por dois tradutores fluentes em inglês e português. Um dos tradutores era fonoaudiólogo e professor de línguas estrangeiras, com conhecimento sobre a temática, permitindo auxiliar nos termos técnicos específicos do conteúdo do instrumento. O outro tradutor era professor de línguas estrangeiras, que deu ênfase nas características linguísticas e nos aspectos mais gerais envolvidos no processo de tradução, como a correspondência entre as versões original e traduzida, além do nível de compreensão do instrumento.

Após essa etapa, tendo as duas versões do instrumento traduzido, juntamente com o instrumento original, foi realizada uma síntese das traduções pela equipe de pesquisadores, composta pelos dois tradutores, a pesquisadora principal e o orientador do estudo. Neste momento, foram consideradas as duas versões das traduções, sendo discutida qual delas melhor se adequava para cada item ou sendo realizado um arranjo das duas traduções.

Depois, o instrumento foi apresentado a um grupo de dezessete juízes fonoaudiólogos, com expertise na área, que foram convidados a participar por contato via e-mail. A partir disto, os dez juízes que aceitaram participar voluntariamente, avaliaram cada item quanto à sua relevância, viabilidade e adequação. A avaliação foi realizada considerando uma escala de resposta com quatro graduações, variável desde a ausência da característica até a sua presença em intensidade acentuada (ex: irrelevante, pouco relevante, relevante e muito relevante). Participaram desta etapa nove mulheres e um homem, sendo que seis deles possuíam a titulação de doutor e quatro de mestre.

Em seguida, na etapa seguinte do estudo, o instrumento foi aplicado com 14 pais de pessoas com T21, sendo 13 mães e um pai, com perfil diverso, buscando testar o instrumento traduzido quanto aos seus aspectos semânticos e nível de compreensão. Nesta etapa, os pais deveriam indicar, em cada item, se compreendiam o item, as instruções e a escala de respostas, também poderiam, em caso negativo, apresentar alguma sugestão de mudança no texto para que este se tornasse mais adequado ao seu vocabulário.

Para isso, o instrumento foi apresentado aos pais por meio de um questionário online, elaborado no *Google Forms*, que foi enviado por mídias sociais (aplicativo de mensagens), juntamente com vídeo de orientações quanto aos procedimentos para análise. Nove pais participaram desta etapa, mas apenas dois deles foram incluídos por realizarem adequadamente a análise no formato online, conforme orientado.

Ainda, devido à dificuldade apresentada pelos pais em realizar a análise no formato online, foi realizada uma coleta de dados presencial. Doze pais foram reunidos em pequenos grupos e orientados quanto aos procedimentos de análise do questionário impresso. A amostra foi eleita por conveniência, tendo sido convidados os pais participantes de um projeto de extensão na área, que tinham seus filhos com a idade considerada para a aplicação do instrumento (compreendida entre um e vinte e um anos).

Depois, foi realizada a retrotradução por um nativo do inglês e falante do português, tradutor profissional, que realizou uma nova tradução do instrumento em português para o inglês, sem ter acesso ao instrumento original. A partir disso, foi realizada uma comparação entre a versão original, a versão traduzida e a versão retrotraduzida, resultando na adaptação de aspectos do protocolo, culminando na versão pré-final do instrumento traduzido e adaptado.

Por fim, essa última versão do instrumento foi apresentada à autora original, juntamente com a versão retrotraduzida, para aprovação. A análise comparativa da versão traduzida e adaptada com a versão retrotraduzida foi realizada pela equipe de pesquisa.

Após a realização dos ajustes necessários, a versão foi aplicada com pais de pessoas com T21, em um estudo-piloto realizado com participantes de um projeto de extensão e com membros de uma associação sem fins lucrativos, ambos voltados para pessoas com T21. Buscou-se avaliar se os itens estavam adequados quanto ao seu significado e ao nível de compreensão, bem como as instruções para a administração.

Esta última etapa foi realizada no formato online, com divulgação realizada em aplicativo de mensagens e envio do instrumento para preenchimento no *Google Forms*. Nesta etapa, 18 pais responderam ao instrumento, porém dois deles foram excluídos por seus filhos não estarem na faixa-etária estabelecida; outros dois foram excluídos por já terem participado do estudo na etapa de análise pela população-alvo, realizando a função de juízes. Sendo assim, foram incluídos 14 pais, sendo 13 mães e um pai.

As referidas etapas foram realizadas primordialmente no formato online, com envio de e-mails para os juízes participantes, preenchimento de formulários virtuais e realização de videoconferências para reuniões de consenso entre os pesquisadores. Durante a realização da etapa de análise do instrumento pelo público-alvo, foi realizada uma coleta de dados presencial, devido às dificuldades encontradas pela amostra em realizá-la virtualmente, sendo realizada na Clínica-Escola de Fonoaudiologia de uma Instituição de Ensino Superior, localizada na cidade de João Pessoa-PB.

### Procedimentos de análise de dados

Foi realizada uma análise qualitativa e descritiva de todo o processo de tradução e adaptação, incluindo todas as etapas desenvolvidas. Em algumas das etapas do estudo, foi realizada também uma análise quantitativa, para consolidar os resultados encontrados nos julgamentos realizados pelos especialistas e pelos pais, bem como os resultados obtidos no estudo piloto, agrupando-os em formato percentual. Ainda, especificamente na etapa de julgamento pelo comitê de juízes especialistas, foi realizada análise da validade de conteúdo dos itens.

A validade de conteúdo mensura o grau de relevância e representatividade dos elementos do instrumento de medida em um construto específico, com objetivo avaliativo. A partir de um instrumento elaborado, é realizada a análise e o parecer de juízes sobre a ferramenta construída. O Índice de Validade de Conteúdo (IVC) abrange uma análise geral (IVC Total) e por item (IVC Individual) da porcentagem/proporcionalidade da opinião de juízes, sendo que, a taxa não deve ser inferior a 0,80 ou 80%. O IVC envolve a média dos IVC-I, em estudos envolvendo seis ou mais juízes recomenda-se IVC-I não inferior a 0,78^(11,12)^.

## RESULTADOS

Ao longo do processo de adaptação transcultural, a versão original do instrumento passou por várias modificações, estando essas representadas em seis versões diferentes, conforme disposto no Quadro 1. A seguir, serão descritos e detalhados os resultados obtidos em cada etapa conduzida no estudo, durante o processo de tradução e adaptação transcultural.

**Quadro 1:** Versões do instrumento

**Versão original**	**Primeira versão (obtida após a síntese das traduções)**	**Segunda versão (obtida após a análise dos especialistas)**	**Terceira versão (obtida após a análise da população-alvo)**	**Quarta versão (obtida por meio da retrotradução)**	**Quinta versão (obtida após a análise da retrotradução)**	**Versão final (obtida após o estudo piloto)**
My child communicates by using (check all that apply):	Minha criança comunica-se usando (assinale todas as opções aplicáveis):	Meu/Minha filho (a) comunica-se usando (assinale todas as opções aplicáveis):	Meu/Minha filho (a) comunica-se usando (assinale todas as opções aplicáveis):	My child communicates through (check all applicable options):	Meu/Minha filho (a) comunica-se usando (assinale todas as opções aplicáveis):	Meu/Minha filho (a) comunica-se usando (assinale todas as opções aplicáveis):
Speech Pictures/Photos	() Fala; () Imagens/Fotos; () Sistema de comunicação de alta tecnologia (tecnologia assistiva); () Língua de sinais; () Placas de comunicação (Comunicação aumentativa/alternativa); () Outro:	() Fala; () Imagens/Fotos; () Sistema de comunicação de alta tecnologia (tecnologia assistiva); () Língua de sinais; () Prancha de comunicação (Comunicação aumentativa/alternativa); () Outro:	() Fala;	() Speech;	() Fala;	() Fala;
High Tech Communication System Sign Language Communication Board			() Imagens/Fotos;	() Images/Photos;	() Imagens/Fotos;	() Imagens/Fotos;
Other:			() Sistema de comunicação de alta tecnologia (tecnologia assistiva);	() High-technology communication systems (assistive technology);	() Sistema de comunicação de alta tecnologia (tecnologia assistiva);	() Sistema de comunicação de alta tecnologia (tecnologia assistiva);
			() Língua de sinais;	() Sign language;	() Língua de sinais;	() Língua de sinais;
			() Prancha de comunicação (Comunicação aumentativa/alternativa);	() Communication boards (augmentative/alternative communication);	() Prancha de comunicação (Comunicação aumentativa/alternativa);	() Prancha de comunicação (Comunicação aumentativa/alternativa);
			() Gestos;	() Gestures	() Gestos;	() Gestos;
			() Outro:	() Other:	() Outro:	() Outro: ____________________________
My child began to speak at about 2 years 3 years 4 years 5 years after 5 years	Meu filho começou a falar por volta dos: () 2 anos () 3 anos () 4 anos ()5 anos ()depois dos 5 anos.	Meu/minha filho (a) começou a falar por volta dos: () 2 anos () 3 anos () 4 anos ()5 anos ()depois dos 5 anos.	Meu/minha filho (a) começou a falar por volta dos: () 1 ano () 2 anos () 3 anos () 4 anos ()5 anos () depois dos 5 anos, () não fala	My child began speaking at around:	Meu/minha filho (a) começou a falar por volta dos: () 1 ano () 2 anos () 3 anos () 4 anos ()5 anos () depois dos 5 anos, () não fala	Meu/minha filho (a) começou a falar por volta dos:
				() 1 year old () 2 years old () 3 years old () 4 years old () 5 years old () after 5 years old () he/she does not speak		() 1 ano () 2 anos () 3 anos () 4 anos () 5 anos () depois dos 5 anos () não fala
On a scale of 1	Em uma escala de 1 à	Em uma escala de 1 a 10, na qual 1 é incompreensível	Em uma escala de 0 a 10, na qual 0 é ausência de fala e 10 é fala completamente compreensível, como você classificaria a fala do seu/sua filho(a)?	On a scale from 0 to 10, in which 0 is the absence of speech and 10 fully intelligible speech, how would you classify your child’s speech?	Em uma escala de 0 a 10, na qual 0 é ausência de fala e 10 é fala completamente compreensível, como você classificaria a fala do seu/sua filho(a)?	Em uma escala de 0 a 10, na qual 0 é ausência de fala e 10 é fala completamente compreensível, como você classificaria a fala do seu/sua filho(a)?
to 10, where 1 is completely unintelligible and	10, na qual 1 é completamente incompreensível e	e 10 é completamente compreensível, como				() 0 () 1 () 2 () 3 () 4 () 5 () 6 () 7 () 8 () 9 () 10
10 is completely	10 é completamente compreensível, como	você classificaria a fala				
intelligible, how	você classificaria a fala	do seu filho(a)?				
would you rate your child's speech?	do seu filho?					
Have you been told that your child has oral motor difficulties? Yes No	Já lhe disseram que seu filho tem dificuldades motoras orais?	Já lhe disseram que seu filho(a) tem dificuldades motoras orais?	Já lhe disseram que seu/sua filho(a) tem dificuldades motoras orais?	Have you ever been told that your child has oral motor difficulties?	Já lhe disseram que seu/sua filho(a) tem dificuldades motoras orais?	Já lhe disseram que seu/sua filho(a) tem dificuldades motoras orais?
		() sim () não	() sim () não	() yes () no	() sim () não	() sim () não
Have you been told that your child has apraxia or dyspraxia? Yes No	Já lhe disseram que seu filho tem apraxia ou dispraxia? () sim () não	Já lhe disseram que seu filho/filha tem apraxia ou dispraxia? () sim () não	Já lhe disseram que seu/sua filho(a) tem apraxia de fala?	Have you ever been told that your child has speech apraxia?	Já lhe disseram que seu/sua filho(a) tem apraxia de fala?	Já lhe disseram que seu/sua filho(a) tem apraxia de fala?
			() sim () não	() yes () no	() sim () não	() sim () não
People who know my child well have difficulty understanding his/her speech	Pessoas próximas ao meu filho têm dificuldade em entender a sua fala	Pessoas que já conhecem o meu/minha filho(a) têm dificuldade em entender a sua fala	Pessoas que já conhecem o meu/minha filho(a) têm dificuldade em entender a sua fala: () sempre () frequentemente () às vezes () nunca	People who already know my child have difficulties understanding his/her speech:	Pessoas que já conhecem o meu/minha filho(a) têm dificuldade em entender a sua fala: () sempre () frequentemente () às vezes () nunca	Pessoas que já conhecem o meu/minha filho(a) têm dificuldade em entender a sua fala:
				() always () often () sometimes () never		() Sempre () Frequentemente
						() Às vezes () Nunca
People who first meet my child have difficulty understanding his/her speech	Pessoas que conhecem o meu filho pela primeira vez têm dificuldade em entender a sua fala	Pessoas que conhecem o meu/minha filho (a) pela primeira vez têm dificuldade em entender a sua fala	Pessoas que conhecem o meu/minha filho (a) pela primeira vez têm dificuldade em entender a sua fala:	People who meet my child for the first time have difficulties understanding his/her speech:	Pessoas que conhecem o meu/minha filho (a) pela primeira vez têm dificuldade em entender a sua fala:	Pessoas que conhecem o meu/minha filho (a) pela primeira vez têm dificuldade em entender a sua fala:
			() sempre () frequentemente () às vezes () nunca	() always () often () sometimes () never	() sempre () frequentemente () às vezes () nunca	() Sempre () Frequentemente
						() Às vezes () Nunca
My child communicates primarily by using speech	Meu filho se comunica principalmente através da fala	Meu filho(a) se comunica principalmente através da fala	Meu/Minha filho(a) se comunica principalmente através da fala:	My child communicates mainly through speech:	Meu/Minha filho(a) se comunica principalmente através da fala:	Meu/Minha filho(a) se comunica principalmente através da fala:
			() sempre () frequentemente () às vezes () nunca	() always () often () sometimes () never	() sempre () frequentemente () às vezes () nunca	() Sempre () Frequentemente
						() Às vezes () Nunca
When someone can't understand my child's speech, family members interpret for him or her	Quando alguém não consegue entender a fala do meu filho, os membros da família a interpretam	Quando alguém não consegue entender a fala do(a) meu/minha filho(a), os membros da família a interpretam	Quando alguém não consegue entender a fala do(a) meu/minha filho(a), os membros da família a interpretam:	When someone does not understand my child’s speech, family members interpret it:	Quando alguém não consegue entender a fala do(a) meu/minha filho(a), os membros da família a interpretam:	Quando alguém não consegue entender a fala do(a) meu/minha filho(a), os membros da família a interpretam:
			() sempre () frequentemente () às vezes () nunca	() always () often () sometimes () never	() sempre () frequentemente () às vezes () nunca	() Sempre () Frequentemente
						() Às vezes () Nunca
In infancy, my child made cooing sounds (single sounds)	Na infância, o meu filho fazia sons murmurando (sons únicos)	Na infância, meu/minha filho(a) produzia sons isolados	Na infância, meu/minha filho(a) produzia sons isolados:	During childhood, my child produced isolated sounds:	Na infância, meu/minha filho(a) produzia sons isolados:	Na infância, meu/minha filho(a) produzia sons isolados (sons individuais, por exemplo: sons de consoantes ou de vogais):
			() sempre () frequentemente () às vezes () nunca	() always () often () sometimes () never	() sempre () frequentemente () às vezes () nunca	() Sempre () Frequentemente
						() Às vezes () Nunca
In infancy, my child babbled strings of sounds	Na infância, o meu filho balbuciava sequências de sons	Na infância, o meu/minha filho(a) balbuciava sequências de sons	Na infância, o meu/minha filho(a) balbuciava sequências de sons:	During childhood, my child babbled sound sequences:	Na infância, o meu/minha filho(a) balbuciava sequências de sons:	Na infância, o meu/minha filho(a) balbuciava sequências de sons (geralmente associando consoante + vogal, ex: ma ma):
			() sempre () frequentemente () às vezes () nunca	() always () often () sometimes () never	() sempre () frequentemente () às vezes () nunca	() Sempre () Frequentemente
						() Às vezes () Nunca
My child had difficulty sucking and swallowing liquids in infancy	Na infância, o meu filho tinha dificuldade em sugar e engolir líquidos	Na infância, meu/minha filho(a) tinha dificuldade em sugar e engolir líquidos	Na infância, meu/minha filho(a) tinha dificuldade em sugar e engolir líquidos:	During childhood, my child had difficulties sucking and swallowing liquids:	Na infância, meu/minha filho(a) tinha dificuldade em sugar e engolir líquidos:	Na infância, meu/minha filho(a) tinha dificuldade em sugar e engolir líquidos:
			() sempre () frequentemente () às vezes () nunca	() always () often () sometimes () never	() sempre () frequentemente () às vezes () nunca	() Sempre () Frequentemente
						() Às vezes () Nunca
My child had feeding difficulties when s/he started eating solid foods	Meu filho teve dificuldades de alimentação quando começou a comer alimentos sólidos	Meu/minha filho(a) teve dificuldades de alimentação quando começou a comer alimentos sólidos	Meu/minha filho(a) teve dificuldades de alimentação quando começou a comer alimentos sólidos:	My child had difficulties eating when he/she started having solid foods:	Meu/minha filho(a) teve dificuldades de alimentação quando começou a comer alimentos sólidos:	Meu/minha filho(a) teve dificuldades de alimentação quando começou a comer alimentos sólidos:
			() sempre () frequentemente () às vezes () nunca	() always () often () sometimes () never	() sempre () frequentemente () às vezes () nunca	() Sempre () Frequentemente
						() Às vezes () Nunca
My child currently has difficulties with swallowing liquids	Meu filho atualmente tem dificuldades em engolir líquidos.	Meu/minha filho(a) atualmente tem dificuldades em engolir líquidos.	Meu/minha filho(a) atualmente tem dificuldades em engolir líquidos:	My child currently has difficulties swallowing liquids:	Meu/minha filho(a) atualmente tem dificuldades em engolir líquidos:	Meu/minha filho(a) atualmente tem dificuldades em engolir líquidos:
			() sempre () frequentemente () às vezes () nunca	() always () often () sometimes () never	() sempre () frequentemente () às vezes () nunca	() Sempre () Frequentemente
						() Às vezes () Nunca
My child currently has difficulties with feeding/eating	Meu filho atualmente tem dificuldades em se alimentar/comer	Meu/minha filho(a) atualmente tem dificuldades em comer	Meu/minha filho(a) atualmente tem dificuldades em comer	My child currently has difficulties eating:	Meu/minha filho(a) atualmente tem dificuldades em comer	Meu/minha filho(a) atualmente tem dificuldades em comer:
			() sempre () frequentemente () às vezes () nunca	() always () often () sometimes () never	() sempre () frequentemente () às vezes () nunca	() Sempre () Frequentemente
						() Às vezes () Nunca
My child had low tone in the muscles of the face (lips, tongue, cheeks) in infancy	Na infância, meu filho tinha pouca força nos músculos do rosto (lábios, língua e bochechas)	Na infância, meu/minha filho(a) tinha pouca força nos músculos do rosto e da boca (lábios, língua e bochechas)	Na infância, meu/minha filho(a) tinha pouca força nos músculos do rosto e da boca (lábios, língua e bochechas):	During childhood, my child had little muscle strength on the face and in the mouth (lips, tongue, and cheeks):	Na infância, meu/minha filho(a) tinha pouca força nos músculos do rosto e da boca (lábios, língua e bochechas):	Na infância, meu/minha filho(a) tinha pouca força nos músculos do rosto e da boca (lábios, língua e bochechas):
			() sempre () frequentemente () às vezes () nunca	() always () often () sometimes () never	() sempre () frequentemente () às vezes () nunca	() Sempre () Frequentemente
						() Às vezes () Nunca
My child currently has low tone in the muscles of the face (lips, tongue, cheeks)	Meu filho atualmente tem baixa força nos músculos da face (lábios, língua, bochechas)	Meu/Minha filho(a) atualmente tem pouca força nos músculos do rosto e da boca (lábios, língua, bochechas)	Meu/Minha filho(a) atualmente tem pouca força nos músculos do rosto e da boca (lábios, língua, bochechas):	My child currently has little muscle strength on the face and in the mouth (lips, tongue, and cheeks):	Meu/Minha filho(a) atualmente tem pouca força nos músculos do rosto e da boca (lábios, língua, bochechas):	Meu/Minha filho(a) atualmente tem pouca força nos músculos do rosto e da boca (lábios, língua, bochechas):
			() sempre () frequentemente () às vezes () nunca	() always () often () sometimes () never	() sempre () frequentemente () às vezes () nunca	() Sempre () Frequentemente
						() Às vezes () Nunca
My child was late (delayed) in beginning to speak	Meu filho começou a falar tarde (atrasado).	Meu/minha filho(a) começou a falar tarde (atrasado).	Meu/minha filho(a) começou a falar tarde (atrasado):	My child began to talk late (delayed):	Meu/minha filho(a) começou a falar tarde (atrasado):	-
			() sempre () frequentemente () às vezes () nunca	() always () often () sometimes () never	() sempre () frequentemente () às vezes () nunca	
My child makes the same speech errors consistently	Meu filho comete consistentemente os mesmos erros de fala	Meu/minha filho(a) comete sempre os mesmos erros de fala	Meu/minha filho(a) comete os mesmos erros de fala:	My child makes the same speech errors:	Meu/minha filho(a) comete os mesmos erros de fala:	Meu/minha filho(a) comete os mesmos erros de fala (por exemplo, omite ou substitui os sons, de forma consistente, como o som de “s” por “t”):
			() sempre () frequentemente () às vezes () nunca	() always () often () sometimes () never	() sempre () frequentemente () às vezes () nunca	() Sempre () Frequentemente
						() Às vezes () Nunca
Sometimes, my child can say a word but at other times, my child has difficulty saying the same word	Às vezes, meu filho consegue falar uma palavra, mas em outras ocasiões, tem dificuldade em falar a mesma palavra	Às vezes, meu/minha filho(a) consegue falar uma palavra, mas em outras ocasiões, tem dificuldade em falar a mesma palavra.	Às vezes, meu/minha filho(a) consegue falar uma palavra, mas em outras ocasiões, tem dificuldade em falar a mesma palavra:	Sometimes, my child can say a word, while at other times he/she has difficulties saying that same word:	Às vezes, meu/minha filho(a) consegue falar uma palavra, mas em outras ocasiões, tem dificuldade em falar a mesma palavra:	Às vezes, meu/minha filho(a) consegue falar uma palavra, mas em outras ocasiões, tem dificuldade em falar a mesma palavra:
			() sempre () frequentemente () às vezes () nunca	() always () often () sometimes () never	() sempre () frequentemente () às vezes () nunca	() Sempre () Frequentemente
						() Às vezes () Nunca
My child is understandable when s/he says single words, but has greater difficulty in conversation	Meu filho é compreendido quando fala palavras isoladas, mas tem maior dificuldade em conversar	Meu/minha filho(a) é compreendido quando fala palavras isoladas, mas tem maior dificuldade em conversar	Meu/minha filho(a) é compreendido quando fala palavras isoladas, mas tem maior dificuldade em conversar:	My child is understood when he/she says isolated words but has greater difficulties in conversations:	Meu/minha filho(a) é compreendido quando fala palavras isoladas, mas tem maior dificuldade em conversar:	Meu/minha filho(a) é compreendido quando fala palavras isoladas, mas tem maior dificuldade em conversar:
			() sempre () frequentemente () às vezes () nunca	() always () often () sometimes () never	() sempre () frequentemente () às vezes () nunca	() Sempre () Frequentemente
						() Às vezes () Nunca
My child uses a few sounds, but does not make many different sounds	Meu filho usa alguns sons, mas não produz muitos sons diferentes	Meu/minha filho(a) usa alguns sons, mas não produz muitos sons diferentes	Meu/minha filho(a) fala alguns sons, mas não produz sons diferentes:	My child makes some sounds but does not produce different sounds:	Meu/minha filho(a) fala alguns sons, mas não produz sons diferentes:	Meu/minha filho(a) fala alguns sons, mas não produz sons diferentes:
			() sempre () frequentemente () às vezes () nunca	() always () often () sometimes () never	() sempre () frequentemente () às vezes () nunca	() Sempre () Frequentemente
						() Às vezes () Nunca
My child can sing the words in songs more clearly than s/he can say them when speaking	Meu filho consegue cantar as palavras das músicas com mais clareza do que quando as fala	Meu/minha filho(a) consegue cantar as palavras das músicas com mais clareza do que quando as fala.	Meu/minha filho(a) consegue cantar as palavras das músicas com mais clareza do que quando as fala:	My child can sing the lyrics of a song more clearly than when he/she speaks them:	Meu/minha filho(a) consegue cantar as palavras das músicas com mais clareza do que quando as fala:	Meu/minha filho(a) consegue cantar as palavras das músicas com mais clareza do que quando as fala:
			() sempre () frequentemente () às vezes () nunca	() always () often () sometimes () never	() sempre () frequentemente () às vezes () nunca	() Sempre () Frequentemente
						() Às vezes () Nunca
My child shows very slow improvement in speech therapy	Meu filho apresenta melhora muito lenta na terapia de fala	Meu/minha filho(a) apresenta melhora muito lenta na terapia fonoaudiológica	Meu/minha filho(a) apresenta melhora muito lenta na terapia fonoaudiológica:	My child improves very slowly in speech-language therapy:	Meu/minha filho(a) apresenta melhora muito lenta na terapia fonoaudiológica:	Meu/minha filho(a) apresenta melhora muito lenta na terapia fonoaudiológica:
			() sempre () frequentemente () às vezes () nunca	() always () often () sometimes () never	() sempre () frequentemente () às vezes () nunca	() Sempre () Frequentemente
						() Às vezes () Nunca
My child seems to be struggling so hard to say words and sounds	Meu filho parece se esforçar muito para produzir palavras e sons	Meu/minha filho(a) parece se esforçar muito para produzir palavras e sons	Meu/minha filho(a) parece se esforçar muito para produzir palavras e sons:	My child seems to make a great effort to produce words and sounds:	Meu/minha filho(a) parece se esforçar muito para produzir palavras e sons:	Meu/minha filho(a) parece se esforçar muito para produzir palavras e sons:
			() sempre () frequentemente () às vezes () nunca	() always () often () sometimes () never	() sempre () frequentemente () às vezes () nunca	() Sempre () Frequentemente
						() Às vezes () Nunca
My child speaks rapidly	Meu filho fala rapidamente	Meu/minha filho(a) fala rapidamente	Meu/minha filho(a) fala rapidamente: () sempre () frequentemente () às vezes () nunca	My child speaks hastily:	Meu/minha filho(a) fala rapidamente: () sempre () frequentemente () às vezes () nunca	Meu/minha filho(a) fala rapidamente:
				() always () often () sometimes () never		() Sempre () Frequentemente
						() Às vezes () Nunca
My child has fluency (stuttering-like) difficulties when speaking	Meu filho tem dificuldades de fluência (como gagueira) quando fala	Meu/minha filho(a) tem dificuldades de fluência (como gagueira) quando fala	Meu/minha filho(a) tem dificuldades de fluência (como gagueira) quando fala:	My child had fluency difficulties (such as stuttering) when he/she speaks:	Meu/minha filho(a) tem dificuldades de fluência (como gagueira) quando fala:	Meu/minha filho(a) tem dificuldades de fluência (como gagueira) quando fala:
			() sempre () frequentemente () às vezes () nunca	() always () often () sometimes () never	() sempre () frequentemente () às vezes () nunca	() Sempre () Frequentemente
						() Às vezes () Nunca
My child has difficulty hearing	Meu filho tem dificuldade de audição	Meu/minha filho(a) tem dificuldade de audição	Meu/minha filho(a) tem dificuldade de audição:	My child has hearing difficulties:	Meu/minha filho(a) tem dificuldade de audição:	Meu/minha filho(a) tem dificuldade de audição:
			() sempre () frequentemente () às vezes () nunca	() always () often () sometimes () never	() sempre () frequentemente () às vezes () nunca	() Sempre () Frequentemente
						() Às vezes () Nunca
My child has more difficulty saying longer words than shorter words	Meu filho tem mais dificuldade em falar palavras longas do que palavras curtas	Meu/minha filho(a) tem mais dificuldade em falar palavras longas do que palavras curtas	Meu/minha filho(a) tem mais dificuldade em falar palavras longas do que palavras curtas:	My child has greater difficulty speaking longer than shorter words:	Meu/minha filho(a) tem mais dificuldade em falar palavras longas do que palavras curtas:	Meu/minha filho(a) tem mais dificuldade em falar palavras longas do que palavras curtas:
			() sempre () frequentemente () às vezes () nunca	() always () often () sometimes () never	() sempre () frequentemente () às vezes () nunca	() Sempre () Frequentemente
						() Às vezes () Nunca
My child has more difficulty speaking when s/he is using longer phrases or sentences	Meu filho tem mais dificuldade em falar quando utiliza frases ou sentenças mais longas	Meu/minha filho(a) tem mais dificuldade em falar quando utiliza frases ou sentenças mais longas	Meu/minha filho(a) tem mais dificuldade em falar quando utiliza frases ou sentenças mais longas:	My child has greater difficulty speaking when he/she uses longer phrases or sentences:	Meu/minha filho(a) tem mais dificuldade em falar quando utiliza frases ou sentenças mais longas:	Meu/minha filho(a) tem mais dificuldade em falar quando utiliza frases ou sentenças mais longas:
			() sempre () frequentemente () às vezes () nunca	() always () often () sometimes () never	() sempre () frequentemente () às vezes () nunca	() Sempre () Frequentemente
						() Às vezes () Nunca
My child has difficulty saying some consonant sounds	Meu filho tem dificuldade em falar alguns sons de consoantes	Meu/minha filho(a) tem dificuldade em falar algumas consoantes	Meu/minha filho(a) tem dificuldade em falar algumas consoantes:	My child has difficulty saying some consonants:	Meu/minha filho(a) tem dificuldade em falar algumas consoantes:	Meu/minha filho(a) tem dificuldade em falar algumas consoantes:
			() sempre () frequentemente () às vezes () nunca	() always () often () sometimes () never	() sempre () frequentemente () às vezes () nunca	() Sempre () Frequentemente
						() Às vezes () Nunca
My child has difficulty saying some vowel sounds	Meu filho tem dificuldade em falar alguns sons de vogais	Meu/minha filho(a) tem dificuldade em falar algumas vogais	Meu/minha filho(a) tem dificuldade em falar algumas vogais:	My child has difficulty saying some vowels:	Meu/minha filho(a) tem dificuldade em falar algumas vogais:	Meu/minha filho(a) tem dificuldade em falar algumas vogais:
			() sempre () frequentemente () às vezes () nunca	() always () often () sometimes () never	() sempre () frequentemente () às vezes () nunca	() Sempre () Frequentemente
						() Às vezes () Nunca
My child often reverses sounds in words (e.g., aminal for animal)	Meu filho frequentemente inverte sons em palavras (por exemplo: aminal para animal)	Meu/minha filho(a) frequentemente inverte sons em palavras (por exemplo: aminal para animal)	Meu/minha filho(a) frequentemente inverte sons em palavras (por exemplo: aminal para animal):	My child often inverts sounds in words (for example, aminal for animal):	Meu/minha filho(a) frequentemente inverte sons em palavras (por exemplo: aminal para animal):	Meu/minha filho(a) inverte sons em palavras (por exemplo: aminal para animal):
			() sempre () frequentemente () às vezes () nunca	() always () often () sometimes () never	() sempre () frequentemente () às vezes () nunca	() Sempre () Frequentemente
						() Às vezes () Nunca
My child has difficulty with the rhythm of speech (speech sounds choppy, or sometimes slow and sometimes fast)	Meu filho tem dificuldade com o ritmo da fala (a fala parece variável, às vezes lenta e às vezes rápida)	Meu/minha filho(a) tem dificuldade com o ritmo da fala (a fala parece variável, às vezes lenta e às vezes rápida)	Meu/minha filho(a) tem dificuldade com o ritmo da fala (às vezes lenta e às vezes rápida):	My child has difficulties with speech rhythm (sometimes slow, sometimes fast):	Meu/minha filho(a) tem dificuldade com o ritmo da fala (às vezes lenta e às vezes rápida):	Meu/minha filho(a) tem dificuldade com o ritmo da fala (às vezes lenta e às vezes rápida):
			() sempre () frequentemente () às vezes () nunca	() always () often () sometimes () never	() sempre () frequentemente () às vezes () nunca	() Sempre () Frequentemente
						() Às vezes () Nunca
My child prolongs vowel sounds	Meu filho prolonga os sons das vogais	Meu/minha filho(a) prolonga os sons das vogais	Meu/minha filho(a) prolonga os sons das vogais:	My child prolongs vowel sounds:	Meu/minha filho(a) prolonga os sons das vogais:	Meu/minha filho(a) prolonga os sons das vogais:
			() sempre () frequentemente () às vezes () nunca	() always () often () sometimes () never	() sempre () frequentemente () às vezes () nunca	() Sempre () Frequentemente
						() Às vezes () Nunca
My child leaves out sounds in words	Meu filho omite sons nas palavras	Meu/minha filho(a) omite sons nas palavras	Meu/minha filho(a) omite sons nas palavras:	My child omits sounds in words:	Meu/minha filho(a) omite sons nas palavras:	Meu/minha filho(a) omite sons nas palavras:
			() sempre () frequentemente () às vezes () nunca	() always () often () sometimes () never	() sempre () frequentemente () às vezes () nunca	() Sempre () Frequentemente
						() Às vezes () Nunca
My child leaves out syllables in words	Meu filho omite sílabas nas palavras	Meu/minha filho(a) não fala algumas sílabas das palavras	Meu/minha filho(a) não fala algumas sílabas das palavras:	My child does not speak some syllables in words:	Meu/minha filho(a) não fala algumas sílabas das palavras:	Meu/minha filho(a) omite algumas sílabas das palavras:
			() sempre () frequentemente () às vezes () nunca	() always () often () sometimes () never	() sempre () frequentemente () às vezes () nunca	() Sempre () Frequentemente
						() Às vezes () Nunca
My child's speech sounds hypernasal (as if it's coming through his/her nose)	Meu filho fala sons hipernasais (como se estivessem saindo pelo nariz).	Meu/minha filho(a) fala sons hipernasais (como se estivessem saindo pelo nariz).	Meu/minha filho(a) fala sons hipernasais (como se estivessem saindo pelo nariz):	My child speaks with hypernasal sounds (as if coming through the nose):	Meu/minha filho(a) fala sons hipernasais (como se estivessem saindo pelo nariz):	Meu/minha filho(a) fala sons hipernasais (como se estivessem saindo pelo nariz):
			() sempre () frequentemente () às vezes () nunca	() always () often () sometimes () never	() sempre () frequentemente () às vezes () nunca	() Sempre () Frequentemente
						() Às vezes () Nunca
My child talks less with people outside of the circle of friends and family	Meu filho fala menos com pessoas de fora do círculo de amigos e família.	Meu/minha filho(a) fala menos com pessoas de fora do círculo de amigos e família.	Meu/minha filho(a) fala menos com pessoas de fora do círculo de amigos e família:	My child speaks less with people outside his/her circle of friends and family:	Meu/minha filho(a) fala menos com pessoas de fora do círculo de amigos e família:	Meu/minha filho(a) fala menos com pessoas de fora do círculo de amigos e família:
			() sempre () frequentemente () às vezes () nunca	() always () often () sometimes () never	() sempre () frequentemente () às vezes () nunca	() Sempre () Frequentemente
						() Às vezes () Nunca
It is hard for my child to imitate a word that I say	É difícil para meu filho imitar uma palavra que eu falo	É difícil para meu/minha filho(a) imitar uma palavra que eu falo	É difícil para meu/minha filho(a) imitar uma palavra que eu falo:	It is difficult for my child to imitate a word I speak:	É difícil para meu/minha filho(a) imitar uma palavra que eu falo:	É difícil para meu/minha filho(a) imitar uma palavra que eu falo:
			() sempre () frequentemente () às vezes () nunca	() always () often () sometimes () never	() sempre () frequentemente () às vezes () nunca	() Sempre () Frequentemente
						() Às vezes () Nunca
My child's speech is easier to understand when s/he is saying familiar words	A fala do meu filho é mais fácil de entender quando ele está dizendo palavras familiares	A fala do meu/minha filho(a) é mais fácil de entender quando ele(a) está dizendo palavras familiares	A fala do meu/minha filho(a) é mais fácil de entender quando ele(a) está dizendo palavras familiares:	It is easier to understand my child’s speech when he/she is saying familiar words:	A fala do meu/minha filho(a) é mais fácil de entender quando ele(a) está dizendo palavras familiares:	A fala do meu/minha filho(a) é mais fácil de entender quando ele(a) está dizendo palavras familiares:
			() sempre () frequentemente () às vezes () nunca	() always () often () sometimes () never	() sempre () frequentemente () às vezes () nunca	() Sempre () Frequentemente
						() Às vezes () Nunca
My child understands more than s/he can say	Meu filho compreende mais do que ele pode falar	Meu/minha filho(a) compreende mais do que ele(a) pode falar	Meu/minha filho(a) compreende mais do que ele(a) pode falar:	My child understands more than he/she can speak:	Meu/minha filho(a) compreende mais do que ele(a) pode falar:	Meu/minha filho(a) compreende mais do que ele(a) pode falar:
			() sempre () frequentemente () às vezes () nunca	() always () often () sometimes () never	() sempre () frequentemente () às vezes () nunca	() Sempre () Frequentemente
						() Às vezes () Nunca
My child may unexpectedly say a word or phrase perfectly, but then s/he can't repeat it	Meu filho pode espontaneamente falar uma palavra ou frase perfeitamente, mas ele não consegue repeti-la	Meu/minha filho(a) pode espontaneamente falar uma palavra ou frase perfeitamente, mas ele(a) não consegue repeti-la	Meu/minha filho(a) pode espontaneamente falar uma palavra ou frase, mas ele(a) não consegue repeti-la:	My child can spontaneously say a word or phrase, but he/she cannot repeat it:	Meu/minha filho(a) pode inesperadamente falar uma palavra ou frase, mas ele(a) não consegue repeti-la:	Meu/minha filho(a) pode inesperadamente falar uma palavra ou frase, mas ele(a) não consegue repeti-la:
			() sempre () frequentemente () às vezes () nunca	() always () often () sometimes () never	() sempre () frequentemente () às vezes () nunca	() Sempre () Frequentemente
						() Às vezes () Nunca
My child has difficulty with grammar	Meu filho tem dificuldade com a gramática	Meu/minha filho(a) tem dificuldade com a gramática	Meu/minha filho(a) tem dificuldade com a gramática:	My child has difficulties with grammar:	Meu/minha filho(a) tem dificuldade com a gramática:	Meu/minha filho(a) tem dificuldade com a gramática:
			() sempre () frequentemente () às vezes () nunca	() always () often () sometimes () never	() sempre () frequentemente () às vezes () nunca	() Sempre () Frequentemente
						() Às vezes () Nunca
My child is frustrated when people don't understand what s/he is saying	Meu filho fica frustrado quando as pessoas não entendem o que ele está falando	Meu/minha filho(a) fica frustrado(a) quando as pessoas não entendem o que ele(a) está falando	Meu/minha filho(a) fica frustrado(a) quando as pessoas não entendem o que ele(a) está falando:	My child gets frustrated when people do not understand what he/she is saying:	Meu/minha filho(a) fica frustrado(a) quando as pessoas não entendem o que ele(a) está falando:	Meu/minha filho(a) fica frustrado(a) quando as pessoas não entendem o que ele(a) está falando:
			() sempre () frequentemente () às vezes () nunca	() always () often () sometimes () never	() sempre () frequentemente () às vezes () nunca	() Sempre () Frequentemente
						() Às vezes () Nunca

Fonte: Autoria própria

### Etapas de tradução e síntese das traduções

Ao final da etapa de tradução, observou-se a presença de uma versão com uma tradução mais literal dos itens e outra com maiores modificações. Na síntese das traduções, foi observada bastante consistência entre as duas traduções, se diferenciando pela maior ou menor correspondência literal com a versão de origem. A síntese foi realizada considerando a versão de cada item mais adaptada ao contexto, o que resultou em um arranjo das duas traduções, representando a primeira versão do instrumento.

### Etapa de análise da síntese pelos juízes

Nessa etapa, identificou-se que a síntese das traduções foi avaliada pela maioria dos juízes, na quase totalidade dos seus itens (97,77%), como muito relevante, muito viável e adequada. A partir dos resultados obtidos, foi verificado um Índice de Validade de Conteúdo (IVC) de 0,97 quanto à relevância e à viabilidade, e de 0,82 quanto à adequação. Apenas um item foi avaliado como inadequado pela maioria dos juízes, devido aos termos utilizados (“sons murmurando/sons únicos”), classificados como de difícil entendimento para os pais, demandando assim uma adaptação que permitisse compreendê-los.

Ainda considerando a análise dos juízes, um aspecto que demonstrou a necessidade de adequações foi a diferenciação do gênero do filho (masculino/feminino). Optou-se por utilizar, na síntese, apenas o gênero masculino, de modo a simplificar a redação dos itens, dispensando assim a realização da concordância diferenciando ambos os gêneros. Porém, foi considerado pelos juízes que isso dificulta a identificação dos pais e o sentimento de pertencimento desenvolvido pela descrição do gênero do seu filho. Diante disso, foi utilizada a diferenciação de gênero em todos os itens.

Cabe esclarecer que, cada sugestão dos juízes foi analisada pela equipe de pesquisadores, observando-se que mesmo alguns itens apresentando taxa IVC-I inferior a 80%, algumas das considerações não se referiam especificamente ao conteúdo do item. Tais considerações relacionavam-se a aspectos gerais, como a diferenciação do gênero do filho do participante, elencada em vários itens; e a substituição da expressão “minha criança”. Diante da idade abrangida pelo instrumento, os filhos dos participantes poderiam ter entre 1 e 21 anos, não estando assim completamente apreendidos com o uso de tal expressão.

Além disso, vale salientar que todos os juízes avaliaram como adequadas as instruções e a clareza do instrumento, já a estrutura foi julgada como inadequada por apenas um deles. Sendo assim, após essa etapa, e a partir de consenso da equipe responsável pela condução do estudo, foram feitas as modificações necessárias.

### Etapa de análise pelo público-alvo

Nesta etapa, dos 44 itens, identificou-se que a expressiva maioria deles (32 itens) foi compreendida por todos os pais. Alguns aspectos que geraram dificuldades, sendo apontada a necessidade de adaptação, foram as perguntas que faziam referência específica à fala, sendo questionada a sua aplicabilidade quando a criança não fala e comunica-se de modo não verbal.

Também houve dificuldade no entendimento de termos técnicos (como dificuldades motoras orais, apraxia, dispraxia, sons isolados e erros de fala), sendo solicitada a definição ou exemplos. Ainda, foi solicitada a definição quanto ao que seria considerado como fala e como período da infância. Todos os participantes julgaram como adequadas as instruções, a clareza e a estrutura do instrumento.

Diante disso, foi realizada reunião de consenso entre a equipe responsável pela condução do estudo, sendo realizadas as modificações pertinentes. Vale destacar que, assim como na etapa anterior, algumas mudanças foram realizadas mesmo sem terem sido especificamente apontadas pelos participantes. Tais modificações foram identificadas como necessárias pela equipe responsável pela condução do estudo, a partir das análises realizadas conjuntamente.

### Etapa de retrotradução (tradução reversa)

Após as modificações realizadas a partir da análise pelo público-alvo, foi obtida uma nova versão do instrumento, a qual foi submetida à retrotradução (tradução reversa) do português para o inglês.

Na análise comparativa realizada entre as versões retrotraduzida e original foram identificadas algumas diferenças em relação aos tempos verbais e utilização de grau comparativo em alguns itens.

Ainda, verificou-se a distinção existente entre os itens “*People who know my child well have difficulty understanding his/her speech*” e “*People who already know my child have difficulties understanding his/her speech*”, que se diferem pela intensidade marcada pelo uso do advérbio de intensidade “*well”* (bem). Na versão em português, tínhamos o uso da expressão “Pessoas que já conhecem o meu/minha filho(a) têm dificuldade em entender a sua fala”, sendo decidido mantê-la pois, desse modo, representaria o contraponto proposto com a expressão disposta no item imediatamente posterior: “Pessoas que conhecem o meu/minha filho (a) pela primeira vez têm dificuldade em entender a sua fala”.

Também, verificou-se a diferença existente entre as expressões “*My child makes the same speech errors consistently*” e “*My child makes the same speech errors*”, sendo mantida a versão adaptada “Meu/minha filho(a) comete os mesmos erros de fala”. Neste item, considerou-se que o advérbio de modo (consistentemente) já poderia ser apreendido pela opção “sempre”, existente na graduação da escala de respostas, estando assim representados os indivíduos que cometem sempre (de modo consistente) os mesmos erros de fala.

Por fim, foi identificada uma necessidade de mudança ao se comparar as sentenças “*My child may unexpectedly say a word or phrase perfectly, but then s/he can't repeat it*” e “*My child can spontaneously say a word or phrase, but he/she cannot repeat it*”, visto que os termos *unexpectedly* (inesperadamente) e *spontaneously* (espontaneamente) não abordam o mesmo conteúdo. Assim, foi realizada apenas a seguinte modificação: Substituição do termo “espontaneamente” por “inesperadamente” na frase “Meu/minha filho(a) pode espontaneamente falar uma palavra ou frase, mas ele(a) não consegue repeti-la”.

### Etapa de estudo-piloto

A idade dos filhos dos participantes, que apresentavam T21, variou de 1,33 a 21 anos, com média de 9,33 anos. Foram identificadas algumas sugestões que indicaram: necessidade de utilizar mais clareza em algumas perguntas; dificuldade de compreensão da expressão “força nos músculos da face” e do termo “apraxia de fala”; respostas que não condizem com a pergunta; modificação na escala de respostas para “sim” ou “não”; diminuição na quantidade de itens; perguntas não aplicáveis para os pais de bebês; perguntas com o mesmo sentido; e necessidade de especificar a idade a qual se refere o termo “na infância”.

A maioria dos participantes (64,28%) não apresentou dificuldade em compreender o significado dos itens e nenhum dos participantes apresentou dificuldade em responder ao questionário no formato online.

Nas sugestões apontadas pelos pais, cabe esclarecer que, elas foram apresentadas individualmente pelos participantes, sem coocorrência, indicando que os aspectos apresentados não foram recorrentes e que, por isso, possivelmente não impactem significativamente na compreensão. Porém, estes aspectos conjuntamente trouxeram prejuízos, conforme evidenciado no percentual de pais que apresentaram dificuldades de compreensão, ainda que considerando uma amostra pequena de participantes.

Diante disso, no que diz respeito à clareza, foi realizada revisão do instrumento e verificada a pertinência em acrescentar exemplos de sons isolados, sequência de sons e erros de fala.

Quanto às indicações de respostas que não condizem com a pergunta, necessidade de modificação na escala de respostas para “sim” ou “não” e de diminuição na quantidade de itens, entende-se que os respondentes apresentaram dificuldades com a utilização da escala de respostas apresentada no estudo. Isso ocorre pois a análise da referida escala se torna mais dificultosa por envolver uma graduação da frequência de ocorrência das características ao invés de uma escolha dicotômica.

Contudo, optou-se por mantê-la por permitir apreender com maior detalhamento o construto avaliado pelo instrumento, bem como, possibilitar a comparabilidade com outros estudos que já utilizaram ou que venham a utilizar o instrumento em sua versão original e versões adaptadas.

No que diz respeito à sugestão de que havia perguntas com o mesmo sentido, foi identificada uma proximidade existente entre os itens “Meu/minha filho (a) começou a falar por volta dos: () 1 ano () 2 anos () 3 anos () 4 anos () 5 anos () depois dos 5 anos, () não fala” e “Meu/minha filho(a) começou a falar tarde (atrasado): () sempre () frequentemente () às vezes () nunca”, visto que a primeira pergunta já permite apreender o conteúdo abordado na segunda.

Bem como, não caberia necessariamente aos pais identificar se a idade de início da fala dos seus filhos foi atrasada, mesmo que possam fazê-lo intuitivamente ao compará-los com outras crianças de idade semelhante ou com a mesma condição clínica ou, ainda, ao ter sido tal característica indicada por um profissional. Também, a escala apresentada no segundo item não é adequada, uma vez que a resposta deveria ser dicotômica (sim ou não). Sendo assim, decidimos por retirar o segundo item.

Quanto aos demais itens e perguntas, julgamos que se referem a nuances específicas da fala, que podem parecer semelhantes para o leigo, mas que para o clínico da linguagem representam análises importantes, que merecem ser consideradas no instrumento.

Por fim, no que se refere à sugestão para especificar a idade a qual se refere o termo “na infância”, tal comentário já havia sido apontado nas etapas de análise pelos especialistas e pelo público-alvo. Diante disso, decidiu-se por delimitar o período, nas orientações prévias do instrumento, considerando os limites estabelecidos pela Organização Mundial de Saúde e adotados pelo Ministério da Saúde na Política Nacional de Atenção Integral à Saúde da Criança, que compreendem a criança como o indivíduo de zero a 9 anos^(13)^.

Também, foi realizada a delimitação do limite de idade (18 anos) a ser considerado para os filhos com T21, cujas características serão investigadas por meio do instrumento, estabelecendo assim uma equivalência com o término da adolescência e início da idade adulta, conforme disposto no Estatuto da Criança e do Adolescente^(14)^.

Sendo assim, a partir do consenso realizado entre os pesquisadores responsáveis pela condução do estudo, foram realizadas as modificações pertinentes, resultando na versão final do instrumento.

## DISCUSSÃO

A tradução e adaptação transcultural, realizada neste estudo, tomou como referência a metodologia proposta por Borsa, Damásio e Bandeira^(9)^, a partir das Diretrizes de Beaton et al.^(15)^ e da *International Test Comission* ^(16)^. Ao longo deste processo, foi identificada uma diversidade de metodologias distintas para condução de estudos em tradução e adaptação de instrumentos, conforme já evidenciado anteriormente em estudo de revisão na área^(17)^.

Nesse contexto, outro estudo de revisão da literatura identificou a variedade de metodologias utilizadas nos estudos de tradução e adaptação de instrumentos em Fonoaudiologia no português brasileiro, que se fundamentam em instruções padronizadas e diretrizes específicas^(18)^.

Tal aspecto dificulta a comparabilidade existente entre as pesquisas e a padronização de procedimentos para a obtenção dos melhores resultados, no que diz respeito à equivalência entre as versões original e adaptada. Diante disso, demonstra-se a necessidade de empreender esforços na elaboração de consenso entre grupos de pesquisadores na área, para sistematização das evidências e padronização de um delineamento metodológico a ser seguido nos estudos em tradução e adaptação de instrumentos clínicos.

Nessa perspectiva, vale salientar a escassez de referências práticas sobre os procedimentos e análises envolvidas na construção e adaptação de instrumentos clínicos^(19)^, aspecto ainda observado atualmente. Por mais que observemos avanços na área, principalmente no contexto da Psicometria (na elaboração e adaptação de instrumentos psicológicos), com a tentativa de elaboração de consenso para orientar os pesquisadores na área, ainda há uma grande diversidade metodológica e faltam evidências para favorecer a escolha de uma delas em detrimento das demais.

Sendo assim, a realização do processo de tradução e adaptação fica, por vezes, baseada na opinião de especialistas e nos procedimentos por eles indicados, considerando a sua experiência prática na área e o conhecimento das diretrizes internacionais. Na Fonoaudiologia, este é um campo rico a ser explorado, para a análise de evidências na área, buscando oferecer instrumentos aos clínicos e favorecer a realização de estudos e comparações transculturais.

Diante do exposto, apresentaremos a seguir algumas considerações sobre o processo de tradução e adaptação desenvolvido neste estudo, comparando os procedimentos adotados com os preconizados pelas diretrizes internacionais e pelos pesquisadores na área.

### Etapas de tradução e síntese das traduções

Beaton et al.^(15)^orientam que o processo de tradução seja realizado por dois tradutores bilíngues independentes, cuja língua materna seja a língua-alvo, sendo um deles um tradutor consciente dos conceitos examinados e outro não consciente (tradutor ingênuo). Os referidos autores também indicam que a síntese das traduções deve ser realizada a partir de um consenso entre os autores e um observador, produzindo uma tradução comum^(15)^.

Já as Diretrizes da ITC^(16)^recomendam a escolha de tradutores, se possível, com experiência no conteúdo do teste e com conhecimento dos princípios de avaliação, além disso, que sejam nativos no idioma e que estejam vivendo naquela cultura. Ainda, recomendam um procedimento de dupla tradução e reconciliação, que visa abordar as deficiências e os riscos de confiar em idiossincrasias de traduções simplificadas. Nessa abordagem, um terceiro tradutor independente, ou um grupo de especialistas, identifica e resolve qualquer discrepância entre as traduções alternativas e as reconcilia em uma única versão^(16)^.

Tais recomendações foram parcialmente seguidas no presente estudo, no qual os dois tradutores eram nativos do português e fluentes no inglês; e ambos os tradutores possuíam conhecimento acerca da temática apraxia de fala na infância, por comporem a equipe de pesquisa, possuírem publicações na área e estarem familiarizados com o construto avaliado. Ainda, a síntese das traduções foi realizada a partir de um consenso envolvendo os dois tradutores e os outros dois pesquisadores participantes do estudo. Logo após, as duas traduções e a síntese delas foram submetidas ao julgamento de juízes especialistas.

A abordagem utilizada, com a escolha por tradutores que conheciam a temática, a análise das traduções pela equipe de pesquisa, realizando conjuntamente as decisões e escolhendo o conteúdo mais adequado para a síntese das traduções, foi bastante satisfatória. Bem como, o julgamento realizado pelos juízes especialistas acerca da adequação da síntese das traduções, a partir do acesso à versão original e às duas traduções, tornou possível uma análise de observadores externos, especialistas no construto avaliado, para estabelecer a equivalência entre as versões original e traduzida, conforme será exposto a seguir.

### Etapa de julgamento pelos juízes especialistas

Beaton et al.^(15)^argumentam que o papel do comitê de especialistas é consolidar todas as versões do questionário e desenvolver o que seria considerada a versão pré-final do instrumento para Teste de Campo. Para isso, a comissão seria responsável por revisar todas as traduções e chegar a um consenso sobre qualquer discrepância.

As Diretrizes da ITC^(16)^ consideram como um “especialista” uma pessoa ou um grupo com conhecimento suficiente: (1) dos idiomas envolvidos, (2) das culturas, (3) do conteúdo do teste, e (4) dos princípios gerais da testagem. Nessa perspectiva, recomendam a compilação de dados de julgamento por revisores, para maximizar a adequação da adaptação do teste para as populações alvo, bem como, a utilização de revisores nativos da cultura e idioma locais para avaliar a tradução/adaptação do teste.

Tais aspectos foram considerados no presente estudo, uma vez que os juízes especialistas possuíam as qualificações para tal, sendo nativos do idioma e cultura-alvo, com conhecimento do conteúdo abordado no teste. Eles analisaram as duas versões propostas na etapa de tradução e realizaram um julgamento de cada item disposto na síntese das traduções, verificando sua adequação, viabilidade e relevância, bem como apresentando as suas sugestões de mudança.

A partir disto, foram realizadas as adequações necessárias, por meio de um consenso da equipe condutora do estudo, que consolidou as modificações apontadas individualmente pelos juízes e realizou a escolha daquelas consideradas pertinentes. Optou-se por utilizar tal metodologia por ser mais viável no contexto de realização do estudo, visto que não seria praticável promover um consenso entre todos os dez juízes que participaram voluntariamente da pesquisa. A metodologia utilizada conseguiu atender aos objetivos propostos no estudo, tendo sido verificada a pertinência, a viabilidade e a adequação das expressões contidas nos itens, bem como a equivalência com a versão original.

### Etapa de análise pela população-alvo

Esta etapa é pouco explorada na literatura existente na área, sendo importante para avaliar se os itens, as instruções e a escala de resposta são compreensíveis para o público-alvo^(9)^.

Nesse contexto, no presente estudo, esta etapa foi conduzida por meio da análise de uma amostra de participantes da população-alvo, sendo fundamental para a adaptação do instrumento, com diversas contribuições pertinentes e relevantes para a sua adequação ao contexto cultural brasileiro. Assim, demonstrou-se de grande valia para a análise da viabilidade do instrumento e do grau de compreensão do conteúdo dos itens. Deste modo, contribuiu para a realização dos ajustes necessários, anteriormente à realização da retrotradução (que permitiria comparar a versão traduzida e adaptada à versão original), sendo indispensável a sua realização neste momento do estudo.

### Etapa de retrotradução

A retrotradução é definida como um tipo de verificação de validade, sendo importante para destacar inconsistências grosseiras ou erros conceituais na tradução, devendo os tradutores desconhecerem os conceitos explorados no instrumento^(15)^.

Uma desvantagem existente no delineamento de retrotradução é que se esse método for implementado em sua forma mais restrita, não se conseguirá nenhuma revisão da versão no idioma de destino^(16)^. Assim, devem ser consideradas as adaptações existentes, que resultam em diferenciações em relação à versão original, diante do novo contexto cultural. Nesse sentido, orienta-se a utilização de delineamentos múltiplos de tradução, como um delineamento de retrotradução para verificar a versão de destino criada por meio de tradução dupla e reconciliação por uma gama de especialistas^(16)^.

No presente estudo, tais diretrizes foram consideradas, a retrotradução foi realizada por um tradutor independente, sem acesso a versão original ou a versão no idioma de destino (traduzida e adaptada). A versão retrotraduzida foi comparada com a versão original, pela equipe de pesquisa, e foram identificadas as inconsistências existentes, dando ênfase às adaptações realizadas ao novo contexto cultural e à manutenção do sentido originalmente proposto no instrumento, para que o conteúdo dos itens fosse preservado.

Vale destacar que o objetivo desta etapa é avaliar em que medida a versão traduzida está refletindo o conteúdo do item, conforme propõe a versão original, podendo ser utilizada como uma ferramenta prática para que o pesquisador que está adaptando o instrumento possa se comunicar com o autor do instrumento original^(9)^. Nesse sentido, é recomendável que os autores da versão original emitam opinião em relação à versão retrotraduzida^(20)^.

### Etapa de estudo-piloto

Pesquisas locais podem ser utilizadas para avaliar o teste, sendo o estudo-piloto considerado como uma estratégia para fornecer evidência de que as instruções do teste e o conteúdo do item têm um significado semelhante para todas as populações-alvo^(16)^. Nesse sentido, é pertinente a aplicação de um pequeno teste da versão adaptada do instrumento, podendo-se empregar não apenas a administração do teste e a análise de dados, mas principalmente realizar entrevistas com os administradores e os examinandos para obter suas críticas ao próprio teste^(16)^.

Idealmente, devem ser testadas entre 30 e 40 pessoas, sendo essa a etapa final do processo de adaptação, com o teste da versão pré-final em sujeitos ou pacientes do público-alvo^(15)^. Nessa perspectiva, uma limitação apresentada no presente estudo foi o número reduzido de participantes no estudo-piloto realizado, uma vez que tivemos apenas quatorze indivíduos. Contudo, a partir das considerações apontadas pelos voluntários, foi possível observar e refletir sobre aspectos não identificados anteriormente, como a possibilidade de juntar itens do instrumento e modificar a escala de respostas, bem como, verificar aspectos já anteriormente citados, realizando os ajustes necessários e obtendo a versão final do instrumento traduzido e adaptado.

## CONCLUSÃO

A tradução e adaptação transcultural permitiu verificar as evidências de validade baseadas no conteúdo da versão brasileira do *Down Syndrome Speech Intelligibility Survey.* O referido instrumento possui relevância para o rastreio da Apraxia de fala associada à Trissomia do Cromossomo 21, na população brasileira, considerando à correspondência existente entre as características nele elencadas e os achados da literatura existente na área. Entretanto, para verificar as suas demais evidências de validade e confiabilidade, será necessário submetê-lo as etapas seguintes do processo de validação.

No contexto da T21, os desafios do diagnóstico diferencial das alterações de fala são ainda maiores, havendo uma dificuldade em separar aquilo que é próprio da condição genética, da apraxia ou de outros transtornos de fala e linguagem, com diversas variáveis interferentes nessa análise.

Acredita-se que, a partir da investigação das características da AFI nesta população, proporcionada pelo instrumento traduzido e adaptado neste estudo, será possível rastrear e posteriormente realizar avaliação diagnóstica especializada destes indivíduos, com detalhamento dos diversos aspectos relacionados ao funcionamento da linguagem expressiva e compreensiva.

O empenho dedicado ao diagnóstico diferencial desta alteração, bem como a investigação pormenorizada dos seus aspectos etiológicos, são fundamentais para que possam ser desenvolvidas intervenções cada vez mais direcionadas aos déficits existentes, de acordo com a condição clínica apresentada. Deste modo, evidencia-se a necessidade de aprofundamento nos estudos na área, de modo a qualificar a assistência, tanto clínica quanto educacional.
